# *Verticillium dahliae* effector Vd06254 disrupts cotton defence response by interfering with GhMYC3‐ *GhCCD8* ‐mediated hormonal crosstalk between jasmonic acid and strigolactones

**DOI:** 10.1111/pbi.70098

**Published:** 2025-04-22

**Authors:** Jianhui Ma, Fan Jiang, Yan Yu, Haodan Zhou, Jingjing Zhan, Jianing Li, Yanli Chen, Ye Wang, Hongying Duan, Xiaoyang Ge, Zhenzhen Xu, Hang Zhao, Lisen Liu

**Affiliations:** ^1^ Henan Normal University Research Base of State Key Laboratory of Cotton Bio‐breeding and Integrated Utilization, College of Life Sciences, Henan Normal University Xinxiang China; ^2^ State Key Laboratory of Cotton Bio‐breeding and Integrated Utilization Institute of Cotton Research of Chinese Academy of Agricultural Sciences Anyang China; ^3^ Key Laboratory of Cotton and Rapeseed (Nanjing), Ministry of Agriculture and Rural Affairs, the Institute of Industrial Crops Jiangsu Academy of Agricultural Sciences Jiangsu China; ^4^ College of Life Sciences Qufu Normal University Qufu China

**Keywords:** *Verticillium dahliae*, Effector, host‐pathogen interaction, cotton, transcription factor

## Abstract

*Verticillium dahliae* is among the most destructive plant pathogens, posing a significant threat to global cotton production. Cotton plants have developed sophisticated immune networks to inhibit *V. dahliae* colonization. Ingeniously, *V. dahliae* employs numerous virulent effectors to surmount plant immune responses. However, the pathogenic mechanisms of *V. dahliae*‐derived effectors remain elusive. In this study, we demonstrate that the Vd06254 effector from *V. dahliae* disrupts the synergistic interaction between jasmonic acid (JA) and strigolactones (SL), thereby suppressing cotton immunity. Ectopic expression of *Vd06254* enhanced susceptibility to both viral and *V. dahliae* infections in *Nicotiana benthamiana* and cotton, respectively. Vd06254 directly interacts with the JA pathway regulator GhMYC3. The nuclear localization signal (NLS) was found to be essential for the virulence of Vd06254 and its interaction with GhMYC3. Additionally, overexpression and knockout of *GhMYC3* in cotton modified the plant's resistance to *V. dahliae*. Our findings further reveal that GhMYC3 inhibits the expression of *GhCCD8* by binding to its promoter, potentially regulating SL homeostasis in cotton through a negative feedback loop. This repression was enhanced by Vd06254, highlighting its crucial role in modulating cotton immunity and illustrating how *V. dahliae* effectors reprogram cotton transcription to disrupt this regulatory mechanism.

## Introduction

The perpetual arms race between microbial pathogens and their host plants has given rise to sophisticated pathogenic and defensive mechanisms (Jone and Dangl, 2006; Zhou and Zhang, 2020). Throughout the interaction between plants and microbial pathogens, plants have evolved an innate immune system characterized by two defence layers (Ngou *et al*., 2022). The first layer is involved in pathogen‐associated molecular patterns (PAMPs)‐triggered immunity (PTI). Conversely, plants have adapted to release R proteins that recognize pathogen effectors, providing the second layer of defence known as effector‐triggered immunity (ETI) (Dodds and Rathjen, 2010). Recent studies have indicated that PTI and ETI reinforce one another, enhancing plant resistance to disease (Ngou *et al*., 2021, 2022; Yuan *et al*., 2021a, 2021b). PTI and ETI can trigger a cascade of immune responses, including transient reactive oxygen species (ROS) burst, mitogen‐activated protein kinase (MAPK) cascades and callose deposition (DeFalco and Zipfel, 2021; de Escocard Azevedo Manhães *et al*., 2021). Additionally, plant tissues inoculated with pathogens generate systemic signals that lead to the distal accumulation of immune‐related hormones such as jasmonic acid (JA) and strigolactones (SL), which play crucial roles in plant defence.

JA is a vital phytohormone that enhances plant resistance to pathogens, including *Verticillium dahliae*, a soil‐borne vascular fungus causing Verticillium wilt in cotton (Cai *et al*., 2009; Dadd‐Daigle *et al*., 2021; Song *et al*., 2020). The scarcity of V. dahliae‐resistant cotton germplasms and the pathogen's evolution significantly threaten cotton production (Xu *et al*., 2011). Upon infection, JA accumulates and is perceived by a complex involving the F‐box protein COI1 receptor and Jasmonate Zim Domain (JAZ) repressor proteins (Sheard *et al*., 2010). In the absence of JA, transcription factors MYC2, MYC3 and MYC4 are competitively inhibited by JAZ cofactors (Pauwels *et al*., 2010; Zhang *et al*., 2015). In response to pathogen infection, the F‐box protein COI1, which binds to the isoleucine conjugate of JA, interacts with SKP1 and CULLIN to form the SCF^COI1^ complex (Wasternack and Strnad, 2018; Gupta *et al*., 2021). This complex recruits JAZ repressor proteins and induces theirs degradation 26S proteasome, thereby alleviating the inhibition of MYC2, which positively regulates the expression of JA signalling defence genes, like *PDF1.2* and *VSP2* (Chini *et al*., 2007; Li *et al*., 2019; Pauwels *et al*., 2010; Thines *et al*., 2007). Studies have shown that JA signal transduction enhances plant resistance to *V. dahliae*, particularly noted by the significant accumulation of JA during infection, which increases the expression of genes involved in JA signal transduction (Fradin *et al*., 2011; Hu *et al*., 2018a). In cotton, the repressor GhJAZ2 weakens the plants' response to JA, thereby preventing the activation of JA‐responsive genes and increasing susceptibility to *V. dahliae* (He *et al*., 2018). Moreover, *GhOPR3*, a critical gene in JA biosynthesis, is phosphorylated by GhCPK33, negatively affecting cotton's resistance to *V. dahliae* (Hu *et al*., 2018b).

SL, a carotenoid‐derived phytohormone isolated from cotton root exudates (Cook *et al*., 1996), is involved in plant growth, development and resistance to biotic and abiotic stresses (Kapulnik *et al*., 2011b; Xiong *et al*., 2020). It has been observed to enhance the resistance of tomato plants to pathogens such as *Botrytis cinerea* and *Alternaria alternata* (Torres‐Vera *et al*., 2014; Xu *et al*., 2019). Generally, the endogenous levels of plant hormones are regulated through feedforward and feedback mechanisms. Notably, antagonism between JA and SL‐signalling pathways has been documented; for example, SL has been shown to increase the susceptibility of rice to root‐knot nematodes by antagonizing JA signalling (Lahari *et al*., 2019). In Sea Island cotton (*Gossypium barbadense* L), expressions of SL biosynthesis genes, *GbCCD7* and *GbCCD8b*, are down‐regulated in roots treated with methyl jasmonate (MeJA) (Yi *et al*., 2023), suggesting that *CCD7* and *CCD8* are likely conserved targets of negative feedback regulation in SL biosynthesis (Yi *et al*., 2023). It has been shown that the JA‐responsive gene *GbMYC2* directly binds to the promoters of *GbCCD7* and *GbCCD8*, thereby repressing their expression through a negative feedback loop (Yi *et al*., 2023). This antagonistic interaction between JA and SL signalling contributes to resistance in cotton against *V. dahliae*. However, the specific molecular mechanisms of antagonism, particularly those influenced by the majority of *V. dahliae* effectors, remain largely unexplored.

Previous studies have shown that *V. dahliae* secretes both apoplastic and intracellular effector proteins, some of which interfere with plant innate immunity by manipulating host target functions and the immune signalling pathways (Liu *et al*., 2021, 2023; Qin *et al*., 2018; Qiu *et al*., 2023; Zhang *et al*., 2017). However, the molecular mechanisms by which the majority of *V. dahliae* effectors regulate host target proteins remain unclear.

In this study, we identified a *V. dahliae* effector named Vd06254, which specifically represses Vd424Y‐triggered cell death and facilitates infection by Potato virus X (PVX) and *V. dahliae* in *planta*. A subset of immune response genes was found to be dysregulated in *Nicotiana benthamiana* plants overexpressing PVX‐Vd06254 and in cotton plants inoculated with *V. dahliae* Vd06254 mutant, indicating that Vd06254 functions as a virulence effector. We demonstrate that GhMYC3 directly binds to the promoter of *GhCCD8* and down‐regulates its expression. Interestingly, Vd06254 enhances the DNA‐binding activity of GhMYC3 to the *GhCCD8* promoter, resulting in further repression of *GhCCD8* expression. Overall, this work reveals a mechanism by which the virulence effector Vd06254 disrupts the crosstalk with JA‐ and SL‐signalling pathways, thus enhancing the susceptibility of cotton to *V. dahliae*.

## Results

### Vd06254 contributes to *verticillium dahliae* infection in plants

**Figure 1:**
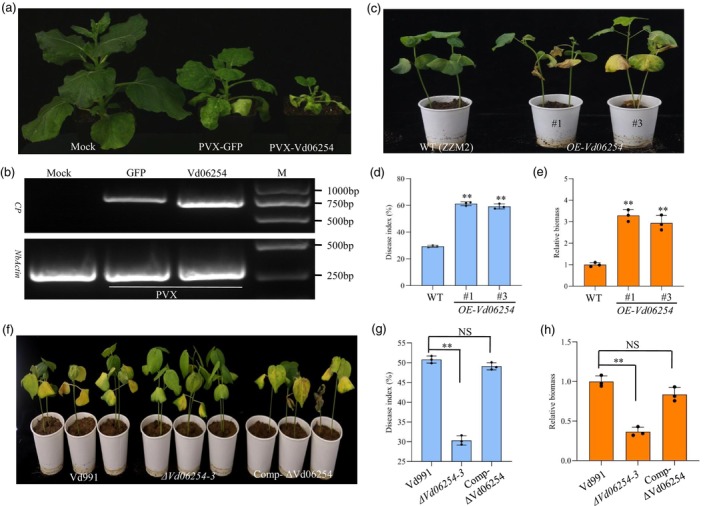
Vd06254 contributes to *Verticillium dahliae* infection in plants. (a) Phenotypic comparison of *Nicotiana benthamiana* plants (*n* = 10) inoculated with PVX*‐GFP*, PVX*‐Vd06254* or mock treatment (no construct as negative control) at 21 days post‐infiltration (dpi). The expression of Vd06254 delayed the normal plant growth and caused the leaves to turn yellow and atrophy. (b) Transcript levels of the Potato virus X (PVX) *CP* gene were assessed using quantitative reverse transcriptase PCR (RT‐PCR). The *NbActin* gene was used as an internal control. (c–e) Disease symptoms (c), disease index (d), the biomass of *Verticillium dahliae* (e) of the wild‐type (WT) (ZZM2, a cotton variety with high resistance to Verticillium wilt) and transgenic cotton plants overexpressing Vd06254 (full length without SP) (*OE‐Vd06254*) at 28 days post‐inoculation (dpi) with *V. dahliae* infection. (f) Disease symptoms of cotton plants (ZM24, a cotton variety with low resistance to Verticillium wilt) inoculated with Vd991, *ΔVd06254‐3*, and Comp‐ ΔVd06254 at 28 dpi. The images were represented by three independent experiments. (g) Disease index of cotton plants leaves at 28 dpi. The disease index (DI) was calculated according to the following formula: DI =100% × (∑representative level × number of diseased leaves at each level)/(total number of investigated leaves × 4). Five levels (0, 1, 2, 3, 4) were classified in seedlings. (h) Relative *V. dahliae* biomass after inoculation by qRT‐PCR. The data shown represent the mean across three biological replicates. Bars indicate standard error. Significance level *P* < 0.01 is represented by **. Experiments were performed three times with similar results.

Effectors proteins that inhibit cell death, secreted by *V. dahliae*, have seldom been reported. In this study, we identified an effector, Vd06254, which inhibits Vd424Y‐induced cell death (Figure S1). Vd06254's suppression of cell death correlates with reduced expression of hypersensitive response (HR)‐specific marker genes, immunity‐related marker genes in hormone pathways, and PTI marker genes when co‐expressed with Vd424Y (Figure S2). To further explore the virulence potential of Vd06254, we employed the virus‐induced virulence effector (VIVE) assay (Shi *et al*., 2020) in *N. benthamiana*. Compared to the mock treatment, *N. benthamiana* plants inoculated with PVX carrying Vd06254 exhibited more pronounced viral symptoms, including wrinkling and necrosis on newly emerged upper leaves (Figure 1a). Concurrently, the transcript levels of the PVX coat protein (*CP*) gene were elevated in plants infected with PVX‐*Vd06254* compared to those infected with PVX carrying *GFP* (PVX‐*GFP*) (Figure 1b). These findings suggest that Vd06254 may act as a virulence factor that enhances viral infection.

To assess the contribution of Vd06254 to *V. dahliae* virulence, we generated transgenic cotton plants expressing *Vd06254* (OE‐Vd06254) in the Zhongzhimian2 (ZZM2) (Figure S3) and evaluated their susceptibility to *V. dahliae* infection. Following 28 days of inoculation, OE‐Vd06254 plants exhibited more severe disease symptoms compared to wild‐type (WT) plants (Figure 1c). Both disease severity and *V. dahliae* biomass in transgenic plants were significantly higher than in WT plants (Figure 1d, e). To further elucidate Vd06254's role in plant infection, we created knock out mutants (*ΔVd06254*) by replacing the *Vd06254* gene with a hygromycin resistance gene in the WT *V. dahliae* strain Vd991 (Figure S4), using previously described methods (Gao *et al*., 2010; Gui *et al*., 2017). Compared to the WT strain Vd991, *ΔVd06254* mutants caused only mild disease symptoms (Figure 1f). Cotton plants inoculated with *ΔVd06254* displayed significantly lower disease indices and reduced biomass compared to those infected with Vd991 (Figure 1g, h). However, complemented ΔVd06254 mutants (Comp‐ ΔVd06254) restored disease symptoms and indices levels comparable to the WT Vd991 (Figure 1f–h). These results strongly suggest that Vd06254 significantly enhances *V. dahliae* virulence. Quantitative reverse transcriptase PCR (RT‐qPCR) further confirmed that the expression levels of selected immune‐associated genes were elevated in cotton roots infected with *ΔVd06254* compared to those infected with Vd991 (Figure S5). Taken together, these findings indicate that Vd06254 is a critical virulence effector necessary for full virulence of *V. dahlia*, enhancing its ability to infect host plants.

### Nuclear localization signal is crucial for the virulence activity of Vd06254

**Figure 2:**
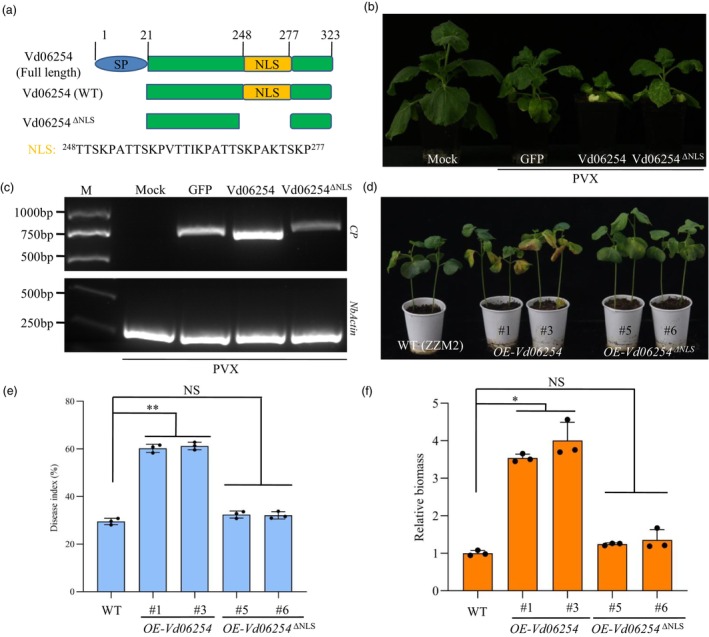
Nuclear localization signal (NLS) of Vd06254 plays a crucial role in *Verticillium dahliae* infection. (a) Schematic diagram of full length (containing signal peptide, SP), wild‐type (WT, full length without SP) and *Vd06254* ^*ΔNLS*^ (NLS deletion) of Vd06254. (b) Phenotypic comparison of *Nicotiana benthamiana* plants (*n* = 10) inoculated with PVX‐*GFP*, PVX‐*Vd06254*, PVX‐*Vd06254* ^*ΔNLS*^ or mock treatment (negative control) at 21 days post‐infiltration (dpi). The plants infected with PVX‐*GFP* or PVX‐*Vd06254* ^*ΔNLS*^ showed milder disease symptoms than those infected with PVX‐*Vd06254*. (c) Transcript levels of the PVX *CP* gene were assessed using quantitative reverse transcriptase PCR (RT‐qPCR). The *NbActin* gene was used as an internal control. (d–f) Disease symptoms (d), disease index (e), the biomass of *V. dahliae* (f) of the WT (ZZM2, a cotton variety with high resistance to Verticillium wilt), transgenic cotton plants overexpressing *Vd06254* (full length without SP) (*OE‐Vd06254*) and *Vd06254* ^*ΔNLS*^ (*OE‐Vd06254* ^*ΔNLS*^) at 28 dpi with *V. dahliae* infection. The data shown represent the mean across three biological replicates. Bars indicate standard error (SE). Significance levels *p* < 0.05 and *p* < 0.01 were represented by * and **, respectively. Experiments were performed three times with similar results.

Domain and motif analysis using the Simple Modular Architecture Research Tool (SMART) database revealed the presence of signal peptide (SP) and an nuclear localization signal (NLS) in Vd06254 (Figure 2a). To explore the role of the NLS in Vd06254's virulence, we generated a mutant, Vd06254 ^ΔNLS^. Intriguingly, the VIVE assay showed that *Vd06254* ^*ΔNLS*^ induced only mild disease symptoms and lower viral accumulation compared to WT *Vd06254*, showing similar results to the *GFP* control (Figure 2b, c). This suggests that NLS of Vd06254 is essential for promoting PVX infection. To further validate the significance of the NLS in controlling *V. dahliae* virulence, we produced heterologous transgenic cotton plants expressing *Vd06254* ^*ΔNLS*^ (OE‐Vd06254^ΔNLS^). These plants exhibited a lower disease index and reduced pathogen biomass compared to OE‐Vd06254 plants (Figure 2d–f). Collectively, these results confirm that the virulence activity of Vd06254 is dependent on its NLS *in planta*, highlight the critical role of nuclear localization in the function of this virulence effector.

### Vd06254 interacts with GhMYC3 in cotton

**Figure 3:**
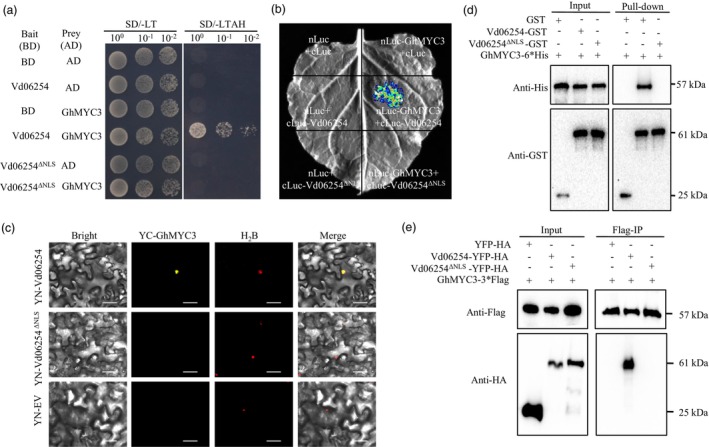
Vd06254 targets GhMYC3 *in vitro* and *in vivo*. (a) Nuclear localization signal (NLS) of Vd06254 mediates its interaction with GhMYC3 in yeast. The yeast strain AH109 was transformed with BD carrying *Vd06254*, *Vd06254* ^*ΔNLS*^ along with AD carrying *GhMYC3*. These transformants were selected on SD medium without Leu and Trp (SD/−Trp/−Leu, SD/−LT) or without Leu, Trp, Ade and His (SD/−Trp/−Leu/−Ade/‐His, SD/‐LTAH). The yeast colonies plates were photographed after four days. Experiments were performed three times with similar results. (b) Vd06254 interacts with GhMYC3 in *Nicotiana benthamiana* by Luciferase complementation imaging (LCI) assay, whereas Vd06254^ΔNLS^ abolished the interactions. *N. benthamiana* leaves were infiltrated with the *Agrobacterium tumefaciens* strain GV3101 carrying the nLuc/cLuc, nLuc‐GhMYC3/cLuc, nLuc/cLuc‐Vd06254, nLuc‐GhMYC3/cLuc‐Vd06254, nLuc/cLuc‐Vd06254^ΔNLS^ and nLuc‐GhMYC3/cLuc‐Vd06254^ΔNLS^ vector combination. Fluorescence signals were detected at 48 h post‐infiltration (hpi). (c) BiFC assay showing the interaction between Vd06254 or Vd06254^ΔNLS^ and GhMYC3. YN, the N terminus of yellow fluorescent protein (YFP); YC, the C terminus of YFP. H_2_B was used as the nuclear localization signal marker with the red fluorescence protein. Scale bars: 50 μm. (d) Vd06254, but not Vd06254^ΔNLS^, interacts with GhMYC3 in vitro pull‐down assay. Vd06254‐GST, Vd06254^ΔNLS^ ‐GST and GhMYC3‐6*His were expressed in *Escherichia coli*. Vd06254‐GST, Vd06254^ΔNLS^‐GST or GST beads were incubated with GhMYC3‐6*His protein. The co‐precipitation was examined via western blotting with anti‐His or anti‐GST antibodies. (e) Co‐immunoprecipitation (Co‐IP) assay confirming that GhMYC3 co‐precipitates with Vd06254 but not Vd06254^ΔNLS^. Total proteins were extracted from *N. benthamiana* leaves expressing YFP‐HA, Vd06254‐YFP‐HA and Vd06254^ΔNLS^‐YFP‐HA with GhMYC3‐3*Flag constructs, respectively. These complexes were immobilized on anti‐Flag beads, and then the co‐precipitates were examined by western blotting with the anti‐HA antibody.

To elucidate the virulence mechanism of Vd06254, we performed a yeast two‐hybrid (Y2H) assay using Vd06254 as bait to screen a cDNA library prepared from cotton plants inoculated with *V. dahliae*. Among the candidates, GhMYC3 emerged as an interacting protein (Figure 3a). Interestingly, mutation of the NLS in Vd06254 abolished the interaction between Vd06254 and GhMYC3 (Figure 3a). To further verify this direct association, we conducted a Luciferase Complementation Imaging (LCI) assay. Constructs of Vd06254, Vd06254^ΔNLS^ and GhMYC3 were fused to the C‐ and N‐terminal halves of the luciferase gene (Luc: cLuc, nLuc), respectively, and co‐expression in *N. benthamiana* leaves through Agrobacterium‐mediated infiltration. After two days, a stronger luciferase signal was observed in leaves co‐expressing nLuc‐GhMYC3 and cLuc‐Vd06254, while the signal was abolished in leaves co‐expressing nLuc‐GhMYC3 and cLuc‐Vd06254^ΔNLS^ (Figure 3b). In line with the Y2H and LCI results, the bimolecular fluorescence complementation (BiFC) assay revealed that YN‐Vd06254, but not Vd06254^ΔNLS^, interacted with YC‐GhMYC3, based on the strong YFP signal and their co‐localization with the nuclear marker H_2_B, in the nucleus of *N. benthamiana* epidermal cells (Figure 3c), consistent with the nuclear localization of Vd06254 and GhMYC3 (Figure S6). The interaction was further confirmed by a glutathione S‐transferase (GST) pull‐down assay *in vitro*. Recombinant GhMYC3 tagged with 6*His was incubated with Vd06254‐GST, Vd06254^ΔNLS^‐GST and GST alone (negative control). GhMYC3‐6*His was specifically pulled down by Vd06254‐GST but not by Vd06254^ΔNLS^‐GST (Figure 3d). Additionally, a co‐immunoprecipitation (Co‐IP) assay was performed *in vivo*. Constructs of YFP‐HA, Vd06254‐YFP‐HA or Vd06254^ΔNLS^‐YFP‐HA were co‐expressed with GhMYC3‐3*Flag in *N. benthamiana* leaves. Total proteins extracted from the leaves were incubated with anti‐Flag magnetic beads. Immunoblotting with anti‐HA antibody revealed significant enrichment of Vd06254‐YFP‐HA, but not YFP‐HA or Vd06254^ΔNLS^‐YFP‐HA (Figure 3e). Collectively, these results demonstrate that the NLS is crucial for the interaction between Vd06254 and GhMYC3, both *in vitro* and *in vivo*. Vd06254^ΔNLS^.

### GhMYC3 enhances cotton resistance against *verticillium dahliae*

**Figure 4:**
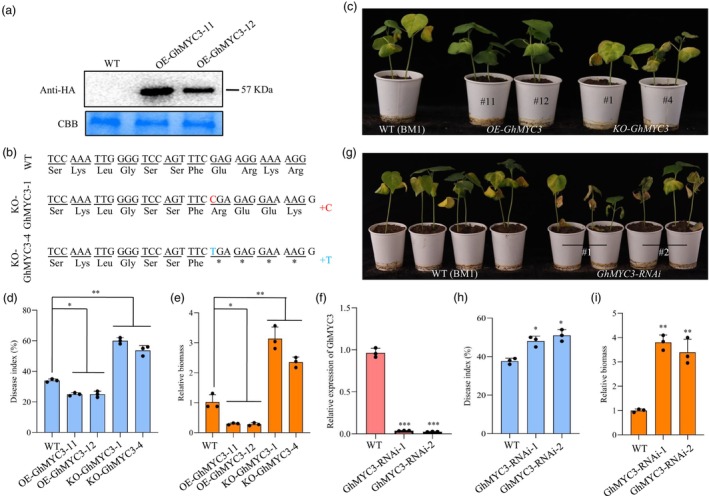
GhMYC3 positively regulates plant defence in cotton. (a) Western blot confirming GhMYC3 production in *GhMYC3*‐overexpressing cotton plants with antibody for the HA. Equal loading was indicated by CBB staining. (b) Knockout of *GhMYC3* patterns, including *KO‐GhMYC3‐1* and *KO‐GhMYC3‐4*. The plus (+) signs indicate the nucleotides inserted at the target sites. (c) Disease symptoms induced with *Verticillium dahliae* on the leaves of wild‐type (WT, BM1), *GhMYC3* overexpressing transgenic cotton plants and *GhMYC3‐*edited cotton plants. Photographs of disease symptoms on cotton were captured at 28 days post‐infection (dpi). (d–e) Disease index (d) and relative *V. dahliae* biomass (e) in infected cotton plants overexpressing *GhMYC3* or edited *GhMYC3* along with the WT at 28 dpi. (f) Expression analysis of *GhMYC3* in *GhMYC3‐RNAi* cotton plants via quantitative reverse transcriptase PCR (RT‐qPCR). (g–i) Disease symptoms (g), disease index (h) and relative *V. dahliae* biomass (i) in infected *GhMYC3‐RNAi* and WT cotton plants at 28 dpi. In D, E, H and I, values represent mean ± standard deviation. * and ** indicate a significant difference at a *P*‐value of <0.05 and <0.01, respectively. Experiments were performed three times with similar results.

To elucidate the role of GhMYC3 in cotton immunity against *Verticillium dahliae*, we overexpressed the *GhMYC3* gene in cotton, generating two transgenic lines (*OE‐GhMYC3‐11*, *OE‐GhMYC3‐12*), which were confirmed to express GhMYC3 via western blot analysis (Figure 4a). When challenged with *V. dahliae*, these overexpressing lines exhibited significantly enhanced resistance compared to WT plants, as evidenced by reduced disease indices and fungal biomass (Figure 4c–e). Subsequently, we employed the CRISPR/Cas9 system to generate *GhMYC3* knockout lines. These edited lines, when challenged with *V. dahliae*, showed decreased resistance, characterized by increased disease indices and fungal biomass compared to WT (Figure 4b–e, Figure S7). Further, to validate *GhMYC3*'s role in plant defence, we suppressed its expression RNA interference (RNAi). RT‐qPCR confirmed a significant reduction in *GhMYC3* transcript levels in the RNAi lines (Figure 4f), which, upon *V. dahliae* challenge, displayed increased susceptibility to infection (Figure 4g), mirroring the phenotype of the knockout lines, with higher disease indices and fungal biomass than WT plants (Figure 4h, i). Collectively, these findings demonstrate that GhMYC3 positively regulates cotton immunity against *V. dahliae*, highlighting its critical function in enhancing plant resistance to this pathogen.

### GhMYC3 down‐regulates *GhCCD8* expression

**Figure 5:**
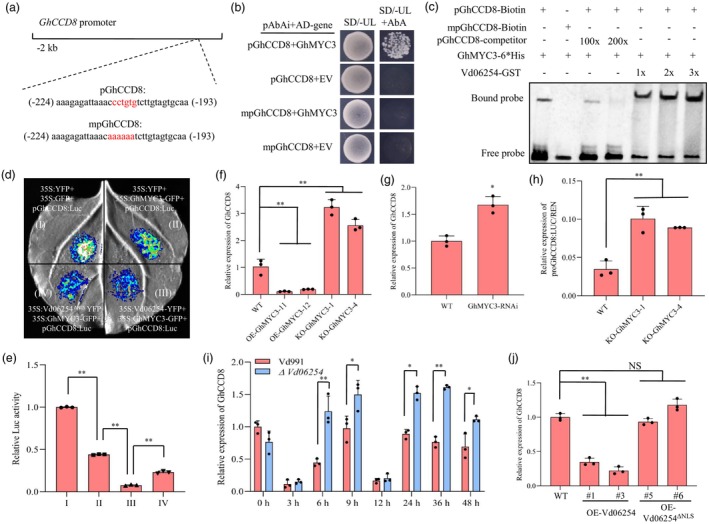
GhMYC3 down‐regulates *GhCCD8* expression. (a) *Cis*‐element bound by GhMYC3 in the upstream of the *GhCCD8* promoter. (b) Yeast one‐hybrid (Y1H) assay showing GhMYC3 binds to the *GhCCD8* promoter. The yeast strain Y1H Gold was co‐transformed with the *pGhCCD8*‐containing pAbAi bait plasmid and AD‐GhMYC3. Yeast transformants were selected on minimal medium containing 100 ng/mL AbA. (c) Verification of the binding of GhMYC3 to the *GhCCD8* promoter and the association among Vd06254, GhMYC3 and *pGhCCD8* via Electrophoretic Mobility Shift Assay (EMSA). The binding capacity of GhMYC3 to the *GhCCD8* promoter enhanced with the increase in Vd06254 concentration. (d, e) Luciferase assay shows that the repressing capacity of GhMYC3 to *GhCCD8* expression is enhanced with the presence of Vd06254 in *Nicotiana benthamiana*. (f, g) Expression levels analysis of *GhCCD8* in leaves of *OE‐GhMYC3* and *KO‐GhMYC3* plants (f), *GhMYC3‐RNAi* plants (g) compared to that in wild‐type (WT) plants via RT‐qPCR. (h) Expression level of pro*GhCCD8*:LUC/REN in *KO‐GhMYC3* plants inoculated with Vd991. (i) Transcript levels in cotton plants leaves overexpressing OE‐Vd06254, OE‐Vd06254^ΔNLS^ or WT plants. In E, F, G, H and I, values represent mean ± standard deviation. * and ** indicate a significant difference at a *P*‐value of <0.05 and 0.01, respectively. Experiments were performed three times with similar results.

A previous study indicated that the GhMYC3 protein binds to the CA T/C GT T/G sequence within the promoters of its target genes, as revealed by DNA Affinity Purification Sequencing (DAP‐Seq) data in cotton (Yuan *et al*., 2023). Notably, the SL biosynthesis gene *GhCCD8* was identified among these targets, with CCTGTG element located upstream of its coding sequence (Figure 5a). To confirm GhMYC3's ability to bind to the *GhCCD8* promoter, a yeast one‐hybrid (Y1H) assays was conducted. Our results showed that GhMYC3 activated the expression of the pAbAi reporter gene driven by the *GhCCD8* promoter, while mutations in the G‐box abolished this binding activity (Figure 5b). Further validation was provided by an Electrophoretic Mobility Shift Assay (EMSA), which confirmed the specific binding of GhMYC3‐6*His protein to the G‐box element of the *GhCCD8* promoter. This interaction was disrupted when mutant probes (Figure 5c). We also performed transient expression assays in *N. benthamiana* using LUC reporters to ascertain if GhMYC3 directly regulates the *GhCCD8* transcription. A 914‐bp sequences of the *GhCCD8* promoter was fused to a *Luc* reporter gene, generating the p*GhCCD8*:Luc vector. Co‐transformation of the 35S:GhMYC3‐GFP effector with the p*GhCCD8*:Luc reporter resulted in significant repression of *Luc* expression compared to the 35S:GFP effector with p*GhCCD8*:Luc reporter (Figure 5d, e), while the sequence alteration in the *GhCCD8* promoter failed to repress expression of p*GhCCD8*:Luc reporter (Figure S8), demonstrating that GhMYC3 directly regulates *GhCCD8* transcription in a G‐box‐dependent manner. Consistent with these findings, the transcript levels of *GhCCD8* were significantly down‐regulated in *GhMYC3*‐overexpressing plants and up‐regulated in *GhMYC3*‐edited lines compared to WT (Figure 5f). Similar trends were observed in *GhMYC3‐RNAi* lines (Figure 5g). Furthermore, transformation of p*GhCCD8*:Luc reporters into GhMYC3 knockout (KO‐GhMYC3) plants under *V. dahliae* inoculation resulted in significant activation of the LUC reporter (Figure 5h). Collectively, these results establish that GhMYC3 acts as a negative regulator of *GhCCD8* expression in cotton, highlighting its role in modulating SL biosynthesis pathways.

### Vd06254 enhances the repressive activity of GhMYC3 on *GhCCD8*

To determine whether Vd06254 influences the GhMYC3‐mediated repression of *GhCCD8* expression, we conducted an Electrophoretic Mobility Shift Assay (EMSA). The results showed that the presence of Vd06254 enhances the DNA‐binding ability of GhMYC3 to the *GhCCD8* promoter (Figure 5c). Additionally, Luc reporter assays revealed that Vd06254 promotes the repressive effect of GhMYC3 on *GhCCD8* expression (Figure 5d, e). Prompted by these findings, we evaluated the expression of *GhCCD8* during the *V. dahliae* inoculation in cotton to determine the impact of Vd06254. Transcript levels of *GhCCD8* were significantly higher in cotton roots infected with the *ΔVd06254* strain compared to those infected with the Vd991 strain (Figure 5i). In contrast, *GhCCD8* transcript levels were reduced in OE‐Vd06254 cotton plants compared to WT and OE‐Vd06254^ΔNLS^ (Figure 5j). Taken together, these results confirm that Vd06254 enhances the repressive activity of GhMYC3 on *GhCCD8*, suggesting a potential mechanism by which *V. dahliae* might manipulate host hormonal pathways to facilitate infection.

### GhCCD8 enhances cotton plant resistance against *verticillium dahliae*

**Figure 6:**
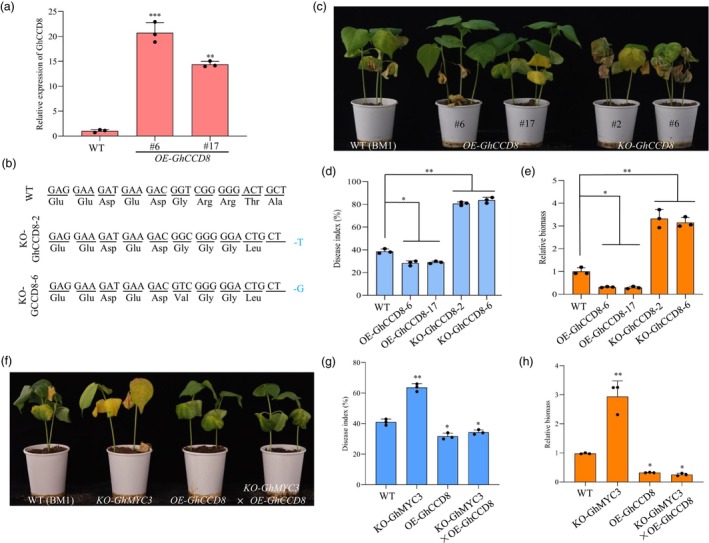
GhCCD8 positively regulates plant defence in cotton. (a) Expression analysis of *GhCCD8* in *OE‐GhCCD8* cotton plants via quantitative reverse transcriptase PCR (RT‐qPCR). (b) Sanger sequencing in wild‐type (WT) cotton Baimian 1 and *GhCCD8*‐edited plants confirming the knockout of GhCCD8. (c) Disease symptoms induced with *Verticillium dahliae* on the leaves of WT, overexpressing *GhCCD8* transgenic cotton plants and *GhCCD8‐*edited cotton plants. Photographs of disease symptoms on cotton were captured at 28 days post‐infection (dpi). (d, e) Disease index (d) and relative *V. dahliae* biomass (e) in infected cotton plants overexpressing *GhCCD8* or edited *GhCCD8* along with the WT at 28 dpi. (f) Presentation of disease symptoms on leaves of WT, *KO‐GhMYC3*, *OE‐GhCCD8* and *KO‐GhMYC3* × *OE‐GhCCD8* transgenic cotton plants following inoculation with *V. dahliae*. Photographs of disease symptoms on cotton were captured at 26 dpi. (g) Disease index (DI) of cotton plants at 26 dpi, assessing disease severity. (h) Quantitative reverse transcriptase PCR (RT‐qPCR) analysis used to measure the relative *V. dahliae* biomass in the stems of infected cotton stems. Values represent mean ± standard deviation; * and ** indicate a significant difference at a *P*‐value of <0.05 and <0.01, respectively. Experiments were performed three times with similar results.

*GhCCD8*, identified as a potential target of GhMYC3, plays a crucial role in the SL biosynthesis pathway. To further investigate the biological roles of *GhCCD8* in cotton resistance to *V. dahliae*, we developed overexpression (*OE‐GhCCD8*) and knockout (*KO‐GhCCD8*) lines (Figure 6a, b) and assessed their resistance to *V. dahliae*. After inoculation with the Vd991 strain, *OE‐GhCCD8* plants exhibited milder Verticillium wilt symptoms compared to WT plants, whereas *KO‐GhCCD8* lines displayed more severe disease symptoms (Figure 6c). Consistently, *OE‐GhCCD8* plants demonstrated lower disease index values and relative fungal biomass, while these parameters were higher in *KO‐GhCCD8* lines compared to WT (Figure 6d, e). These results indicate that *GhCCD8* acts to suppress the progression of *V. dahliae* infection in cotton.

### *GhCCD8* functions genetically downstream of GhMYC3 to augment resistance against *verticillium dahliae* in cotton

To elucidate the genetic interplay between *GhMYC3* and *GhCCD8* in enhancing cotton's resistance to *V. dahliae*, we created hybrid progeny by crossing transgenic lines with these genes knocked out and overexpressed, respectively, yielding *KO‐GhMYC3* × *OE‐GhCCD8* hybrids. Phenotypic evaluations of these hybrids showed milder disease symptoms, lower disease indices and relative biomass compared to WT, mirroring the phenotype observed in *OE‐GhCCD8* transgenic plants (Figure 6f‐h). This suggests that GhMYC3 enhances disease resistance through its regulation of *GhCCD8*, highlighting a strategic interaction within the plant's genetic framework to combat *V. dahliae* infection.

## Discussion

Verticillium wilt, a devastating vascular disease caused by the soil‐borne fungus *V. dahliae*, significantly impacts cotton production. This pathogen deploys various effectors to disrupt host plant immunity through multiple regulatory mechanisms (Qin *et al*., 2018; Wang *et al*., 2019; He *et al*., 2020; Liu *et al*., 2021; Liu *et al*., 2023). Our study demonstrated that the *V. dahliae* effector Vd06254 compromises plant immunity by interfering with the JA‐responsive regulator GhMYC3 and reducing the expression of the SL biosynthesis gene *GhCCD8*. The effectiveness of Vd06254 and effectors like it often hinges on their ability to localize within host cells, a function enabled by features such as the NLS.

During evolution, NLS has been a critical feature in pathogenic microbial effectors, controlling their virulence and influencing host immunity. For example, PSR1, a *Phytophthora sojae* effector, suppresses RNAi‐mediated gene silencing in plants by inhibiting PINP1‐mediated small RNA accumulation to promote infection. Interestingly, PSR1M, the mutant lacking the NLS, largely lost its ability to suppress RNA silencing and pathogenicity (Qiao *et al*., 2013, 2015). Similarly, PsAvh113 promotes infection by relying on its nuclear localization *in planta* (Zhu *et al*., 2023). Conversely, NLS presence in effectors can also induce plant immunity; for example, VdSCP7‐mediated induction of plant immunity depends on its NLS for translocation from the apoplastic space to the nucleus (Zhang *et al*., 2017). Vd424Y also targets the host nucleus to regulate effector‐triggered immunity, relying on its NLS (Liu *et al*., 2021). Our data revealed that Vd06254 harbours an NLS, which is essential for its virulence activity and its interaction with GhMYC3, highlighting the pivotal role of NLS in the functionality of microbial effectors within host cells.

Plants deploy various resistance mechanisms against *V. dahliae*, notably through the synergistic action of phytohormones (Song *et al*., 2020; Yi *et al*., 2023). For instance, abscisic acid (ABA) impedes pathogen diffusion by triggering stomatal closure to prevent pathogen entry and promoting callose deposition (Hawage K *et al*., 2020). The interplay between ABA and JA is particularly crucial; ABA‐deficient and ABA‐insensitive mutants such as *aba2‐12* and *abi4‐1* exhibit compromised JA synthesis and heightened disease susceptibility, highlighting ABA's role in activating JA‐dependent defence responses (Adie *et al*., 2007; Anderson *et al*., 2004). In cotton roots infected with *V. dahliae*, there is a marked increase in JA and ABA levels, along with the upregulation of genes involved in their biosynthesis (Yi *et al*., 2023). Moreover, *V. dahliae* infection significantly elevates SL content in infected roots (Figure S9), and exogenous application of rac‐GR24 mitigates Verticillium wilt symptoms (Figure S10). Previous studies have established that JA enhances plant resistance to pathogens by modulating SL homeostasis through the downregulation of *GbCCD7* and *GbCCD8b* expression (Yi *et al*., 2023).

**Figure 7:**
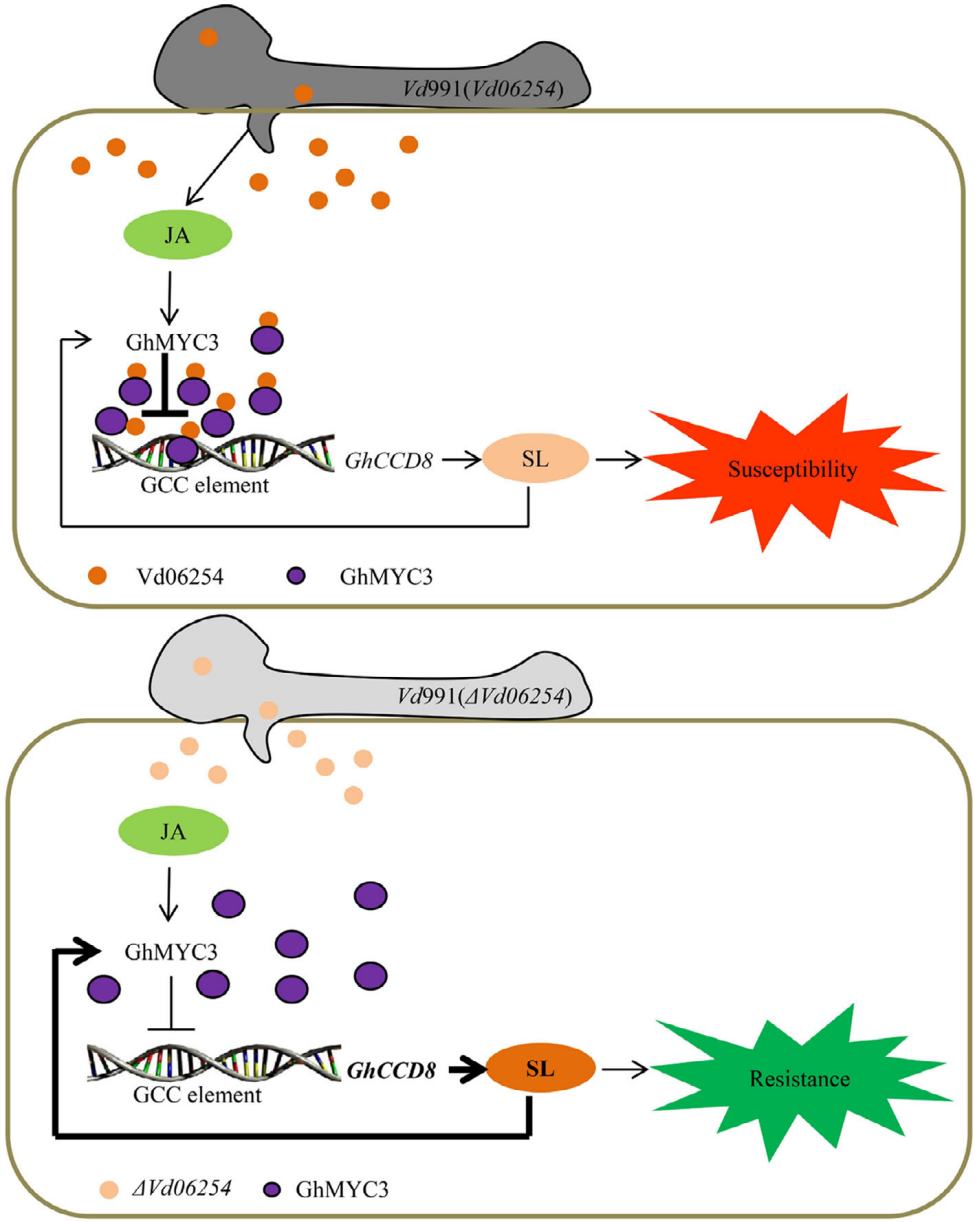
A simplified working model of Vd06254 functions in reprogramming cotton transcriptional during *Verticillium dahliae* infection. In the absence of Vd06254, GhMYC3 binds to the GCC element in the promoter of *GhCCD8* during *ΔVd06254* strain infection, thereby activating cotton immunity with the homeostasis between jasmonic acid (JA) and strigolactones (SL) via a negative feedback loop. In the presence of Vd06254, Vd06254 enhances the repressing capacity of GhMYC3 to *GhCCD8*, which interrupts the GhMYC3‐*GhCCD8*‐mediated negative feedback loop, thereby suppressing the homeostasis between JA and SL.

Our study elaborates on this hormonal interplay, showing that the core JA signalling component GhMYC3 cooperates with the SL synthesis‐related gene *GhCCD8* to enhance resistance against Verticillium wilt in cotton. This interaction forms a hormone synergy model, GhMYC3‐GhCCD8, which regulates the contents of JA and SL, and signal pathways marker genes expression (Figure S12 and S13), resulting in boosting resistance. Application of MeJA reduces *GhCCD8* expression (Figure S11a) and GhMYC3 represses the SL content and SL‐signalling pathway marker genes expression (Figure S13), suggesting that JA disturbs SL homeostasis, which influences cotton's resistance against Verticillium wilt. Conversely, GhCCD8 up‐regulates *GhMYC3* expression during *V. dahliae* infection, enhancing resistance (Figure S11b). What is more, GhCCD8 promotes the JA synthesis (Figure S12b) and induces the expression of JA‐responsive genes *GhVSP2* and *GhPDF1.2* (Figure S12b). Furthermore, the regulation of GhMYC3 by GhCCD8 is likely indirect, as GhCCD8 functions as a synthetase rather than a transcription factor. The intricate regulatory relationship between GhCCD8 and GhMYC3 will be elucidated in our future work. Thus, GhMYC3 functions as a dual transcriptional regulatory factor in regulating cotton responses to Verticillium wilt, a function supported by previous findings (Sasaki‐Sekimoto *et al*., 2013). These results underscore a negative feedback loop between GhMYC3 and GhCCD8 in regulating Verticillium wilt resistance. It is likely that such a negative feedback loop, mediated by GhMYC3 and GhCCD8, enhances cotton plants in finely tuning their defence mechanisms on hormonal levels to cope with *V. dahliae* infection, as negative feedback loops play a vital role in specific signalling nodes and maintaining signalling network homeostasis (Brandman and Meyer, 2008). Sequence analysis further differentiates GhMYC3 from GbMYC2, though both regulate *GhCCD8*/*GbCCD8* expression through a negative feedback loop, thereby enhancing cotton's resistance to Verticillium wilt (Figure S14). Furthermore, our unpublished data suggest that JA regulates ABA‐signalling‐mediated resistance to Verticillium wilt, and Yi *et al*. (2023) reported that SL may regulate ABA signalling to bolster cotton's defence. These insights collectively highlight the critical balance among these hormones in improving disease resistance in cotton. Despite these mechanisms, *V. dahliae* undermines cotton defence to facilitate invasion and colonization. Following colonization, the pathogen secretes various effectors to manipulate the cotton immune response. This study identifies Vd06254 as a virulence effector that suppresses immunity through the GhMYC3‐*GhCCD8* module, mediating hormonal interplay between JA and SL (Figure 7).

Understanding the molecular functions of virulence effectors in modulating plant defence responses is crucial for elucidating plant–pathogen interactions and defining the roles of their regulatory targets in combating disease. Our study provides compelling evidence that the *V. dahliae* effector Vd06254 suppresses the SL‐signalling pathway by inhibiting the expression of the key immune gene *GhCCD8*, which contains GCC elements in its promoter. Notably, Vd06254 promotes the repressive activity of GhMYC3 on *GhCCD8* expression (Figure 5d, e) and directly reduces *GhCCD8* gene expression (Figure 5i). These findings highlight the significant role of GCC elements in the promoter regions of immune genes in regulating transcriptional activation, a process tightly controlled by pathogen virulence effectors during infection. Consequently, our work, in conjunction with previous studies, provides a theoretical foundation for developing durable resistance in cotton against Verticillium wilt by using gene‐editing technologies to modify specific nucleotides within the promoters of immune‐associated genes.

## Materials and methods

### Plant and strain growth conditions

Upland cotton (*Gossypium hirsutum*) varieties Baimian 1 (BM1), Zhongzhimian 2 (ZZM2) and Zhongmiansuo 24 (ZM24) were used in this study. Cotton seedlings were grown in a greenhouse under a 16/8 h light/dark photoperiod, at 25 °C with 75% relative humidity. Transgenic lines overexpressing and knocking out *GhMYC3* and *GhCCD8* were generated in the BM1 background using plant tissue culture technology with vectors WMV068 and WMC016 (Ge *et al*., 2023). The *Vd06254* and *Vd06254* ^*ΔNLS*^ overexpression lines were generated in the ZZM2 background using the WMV067 vector, following the same approach as described in Ge *et al*. (2023). *N. benthamiana* plants were also grown under a 16/8 h light/dark cycle at 22 °C in a greenhouse. *Escherichia coli* strain DH5α was cultured on LB medium at 37 °C with appropriate antibiotics, while *Agrobacterium tumefaciens* strains EHA105, GV3101 and GV3101 (pSoup) were cultured on LB medium at 28 °C, supplemented with appropriate antibiotics.

### Cultivation and infection assay of *verticillium dahliae* strain Vd991

The *V. dahliae* strain Vd991 was initially cultured on potato dextrose agar (PDA) plates, followed by cultivation in Czapek's medium with shaking at 150 rpm and 25 °C until reaching a concentration of approximately 10^8^–10^9^ spores/mL. The harvested fungal spores were then diluted to the desired concentration with distilled water (ddH_2_O). Four‐week‐old WT and transgenic cotton lines were inoculated with 10 mL of conidial suspensions, following the procedure described by Gong *et al*. (2017). The disease index (DI) was calculated following the previously described protocols (Gong *et al*., 2017). Specifically, the DI for cotton plants was calculated using the following formula: DI (%) = [(∑disease grades × number of infected plants) / (total number of checked plants × 4)] × 100. Cotton seedlings were classified into five severity levels (i.e. grades 0, 1, 2, 3 and 4) according to the symptom severity observed on cotyledons and true leaves.

### Yeast one/two‐hybrid assays

The Y1H assay was performed to validate that GhMYC3 was able to bind to the GhCCD8 promoter using the Matchmaker® Gold Yeast One‐Hybrid system (Takara, Tokyo, Japan). A 329‐bp fragment of the *GhCCD8* promoter (pGhCCD8) and a mutant version (mpGhCCD8), where a sequence was replaced by ‘aaaaaa’ were cloned into a yeast vector containing the *AbA* reporter gene to generate constructs pAbAi‐pGhCCD8 or pAbAi‐mpGhCCD8, respectively. The coding region of *GhMYC3* was cloned into the pGADT7 vector by homologous recombination to generate AD‐*GhMYC3*. Constructs containing either pAbAi‐pGhCCD8 or pAbAi‐mpGhCCD8 were transformed into the Y1H Gold yeast cells by the PEG/LiAc method. Subsequently, AD‐GhMYC3 or an empty AD vector was introduced into yeast cells containing pAbAi‐pGhCCD8 or pAbAi ‐mpGhCCD8, and positive transformants were selected on SD/‐Ura/‐Leu (SD/‐UL) medium supplemented with 200 ng/mL Aureobasidin A. The Y2H assay was carried out as previously described (Zhan *et al*., 2021).

### Luciferase complementation imaging assay (LCI)

The LCI assay was performed to verify the interaction between Vd06254 and GhMYC3 in *N. benthamiana* leaves. The full‐length coding sequence of Vd06254, excluding the signal peptide (SP) and its NLS mutant (Vd06254^ΔNLS^), was inserted into the N‐terminal region of the *Luc* reporter vector to generate constructs cLuc‐Vd06254 and cLuc‐Vd06254^ΔNLS^. The coding sequence of GhMYC3 was cloned into the C‐terminal region of the *Luc* reporter vector, yielding nLuc‐GhMYC3. *Agrobacterium tumefaciens* cells harbouring these vectors were co‐infiltrated into *N. benthamiana* leaves. After two days post‐infiltration (dpi), fluorescence signals in the leaves, sprayed with 0.2 mg/mL Luc substrate, were detected using the Tanon 5200 Multi‐chemiluminescent imaging system (Tanon, Shanghai, China).

### Bimolecular fluorescence complementation (BiFC) and subcellular localization assays

The full‐length coding regions of Vd06254, Vd06254^ΔNLS^ and GhMYC3 were individually cloned to the Gateway entry vector QBV3. For the BiFC assay, the QBV3‐Vd06254 and QBV3‐ Vd06254^ΔNLS^ were then transferred into the expression vector pEG201, fusing to the N‐terminal halves of the yellow fluorescence protein (YFP), generating constructs YN‐Vd06254 and YN‐ Vd06254^ΔNLS^; and the QBV3‐GhMYC3 was then transferred into the pEG202 vector, fusing to the C‐terminal halves of the YFP, generating constructs YC‐GhMYC3. For the subcellular localization assay, QBV3‐Vd06254, QBV3‐Vd06254^ΔNLS^ and QBV3‐GhMYC3 were transferred into the expression vector pEG101 (Earley *et al*., 2006; Qiao *et al*., 2013), generating constructs Vd06254‐YFP, Vd06254^ΔNLS^‐YFP and GhMYC3‐YFP. *A. tumefaciens* strain GV3101 cells harbouring the recombinant plasmids (YN‐Vd06254+ YC‐GhMYC3, YN‐Vd06254^ΔNLS^+ YC‐GhMYC3 and YN‐EV + YC‐GhMYC3; Vd06254‐YFP, Vd06254ΔNLS‐YFP and GhMYC3‐YFP) were infiltrated into 4‐week‐old *N. benthamiana* leaves using a previously described protocol (Qiao *et al*., 2015). 48 h post‐infiltration, the YFP signal was detected using an LSM780 confocal microscope (Leica, Wetzlar, Germany).

### Protein extraction and western blot

Proteins were extracted from transgenic cotton lines and *N. benthamiana* leaves infiltrated with *agrobacterium* at two dpi. Collected leaves from transgenic cotton seedlings or infiltrated *N. benthamiana* leaves were ground in liquid nitrogen, then mixed with an equal volume of cold protein isolation buffer comprising 1 mM EDTA (PH 8.0), 20 mM Tris–HCl (PH 7.5), 5 mM dithiothreitol, 150 mM NaCl, 0.1% sodium dodecylsulfate (SDS), 10% glycerol, 1x protein inhibitor cocktail and 1x phosphatase inhibitor cocktail (Roche, Badel, Switzerland) using a vortex homogenizer. The mixture was denatured by boiling for 5 min, cooled on ice for 2 min and centrifuged at 12 000 rpm for 5 min. The supernatant was collected for western blotting analysis to detect positive transgenic cotton seedlings and assess transient expression in *N. benthamiana*. For western blotting, target proteins tagged with specific markers were separated on a 10% Bis‐Tris system denaturing prefabricated gel (Sangon Biotech, Shanghai, China) at 90 V for 2 h, then transferred to the polyvinylidene difluoride (PVDF) membrane (Merck Millipore, Cork, Ireland) at 200 mA for 2 h. The membrane was blocked with 5% skim milk for 1.5 h at room temperature with shaking, then incubated overnight at 4 °C with primary antibodies, including anti‐HA (1:5000; MBL, Tokyo, Japan) or anti‐Flag (1:5000; MBL). This was followed by incubation with an anti‐mouse IgG (H + L chain) pAb‐HRP secondary antibody (1:5000; MBL) for 1.5 h at room temperature. After incubation, the membrane was washed three times with TBST (10 min each). Signals were visualized by adding Immobion Western Chemiluminescent HRP Substrate (Millipore, Burlington, MA, USA) and captured using the Chemidoctm XRS+ system (Bio‐Rad, Hercules, CA, USA).

### Glutathione S‐transferase pull‐down assay

The coding sequences of *Vd06254*, *Vd06254* ^*ΔNLS*^ and *GhMYC3* were cloned into the pGEX‐4 T‐2 vector and pET28a vector using specific primers (Table S1), generating Vd06254‐GST, Vd06254^ΔNLS^‐GST and GhMYC3‐His constructs, respectively. These constructs were then introduced into *E. coli* BL21 competent cells for protein expression. Vd06254‐GST, Vd06254^ΔNLS^‐GST and GST proteins were purified using glutathione agarose. Each of them was incubated with purified GhMYC3‐His using Ni^+^ agarose. The protein complexes from the three combinations were subsequently eluted with glutathione solution. The pulled‐down proteins were detected by western blotting using anti‐His‐tag or anti‐GST‐tag mouse antibodies.

### Co‐immunoprecipitation assay

The coding sequences of Vd06254 and its NLS mutant Vd06254^ΔNLS^ were cloned into the entry vector QBV3 to generate QBV3‐Vd06254 and QBV3‐Vd06254^ΔNLS^ constructs. The coding sequences of GhMYC3 were cloned into QBV3‐3*Flag, generating QBV3‐GhMYC3‐3*Flag. These constructs were subsequently inserted into the pEarlyGate101‐HA and pEarlyGate100 vectors by using the Gateway™ LR Clonase™ II Enzyme Mix (Thermo Fisher Scientific, Waltham, MA, USA), producing pEarlyGate101‐Vd06254‐YFP‐HA, pEarlyGate101‐Vd06254^ΔNLS^‐YFP‐HA and pEarlyGate100‐GhMYC3‐3*Flag. The resulting vectors were introduced into *A. tumefaciens* strain GV3101, which was then cultured at 28 °C with shaking. The cultures were combined and resuspended in MMA buffer (0.2 μM acetosyingone, 1 mM MgCl_2_, 10 mM MES) to an OD_600_ of 1.0, measured using a BioTek Synergy HT microplate reader (BioTek Instruments, Winooski, VT, USA). Total proteins were extracted from *N. benthamiana* infiltrated with *A. tumefaciens* GV3101 strains carrying combinations of Vd06254‐YFP‐HA with GhMYC3‐3*Flag, Vd06254^ΔNLS^‐YFP‐HA with GhMYC3‐3*Flag, and YFP‐HA with GhMYC3‐3*Flag at 2 dpi. The extracts were incubated with FLAG beads (M185‐10R, MBL), and the eluted proteins were detected by immunoblotting with anti‐FLAG (M20008S, MBL) or anti‐HA (M180, MBL) antibodies.

### Electrophoretic mobility shift assay

Recombinant GST‐tagged Vd06254 and His‐tagged GhMYC3 constructs were introduced into *E. coli* BL21 expression cells for protein production. Protein expression was induced by adding 0.2 mM IPTG, followed by incubation at 37 °C for 2 h and then 15 °C overnight with shaking at 180 rpm. The cells were collected and sonicated using a Cole‐Parmer® ultrasonic processor (Cole‐Parmer, Vernon Hills, IL, USA) until the solution was clear. After centrifugation, the supernatant was collected and the recombinant proteins were purified using Ni^+^ beads (Thermo Fisher Scientific). The DNA fragments of *pGhCCD8* or *mpGhCCD8* were end‐labelled with biotin. For DNA competition assays, a non‐labelled DNA fragment was used as the competitor DNA. The EMSA was carried out using the LightShift Chemiluminescent EMSA kit (Thermo Fisher Scientific). Primers utilized for the synthesis of probes used for the EMSA assay are listed in Table S1.

### Quantitative reverse transcriptase PCR

Total RNA was isolated using the FastPure Plant Total RNA Isolation Kit (Vazyme Biotech, Nanjing, Jiangsu, China) and amplified in a 20‐μL reaction with Hiscript II Q RT SuperMix (Vazyme Biotech) for reverse transcription to analyse gene expression. RT‐qPCR assays were performed with ChamQ Universal SYBR qPCR Master Mix (Vazyme Biotech, China) on the ABI QuantStudio 6 Flex biosystem (Thermo Fisher Scientific). *GhActin* in cotton and *NbActin* in *N. benthamiana* served as internal controls. Relative transcriptional levels were calculated using the 2^−ΔΔ*CT*^ method (Livak and Schmittgen, 2001). All reactions included three biological and technical replicates. Primer sequences for the experiments are listed in Table S1.

### Potato virus X assay

Truncated cDNA fragments of *Vd06254* and its mutant Vd06254^ΔNLS^, excluding the N‐terminal signal peptides, were amplified using specific primers (Table S1). The PCR products were cloned into the pGR106 vector, containing the PVX genome. Recombinant plasmids were then introduced into *A. tumefaciens* strain GV3101 containing the pSoup plasmid. The transformed *A. tumefaciens* cells were used to infiltrate the epidermal cells of 3‐week‐old *N. benthamiana* plants following established protocols (Qiao *et al*., 2013). Total RNA was extracted from plants infected with PVX‐GFP, PVX‐Vd06254 and PVX‐Vd06254^ΔNLS^ mutants at 21 dpi. Viral RNAs were detected using probes specific to the PVX *CP* gene (Qiao *et al*., 2013).

### Measurement of JA and SL levels

Cotton roots were harvested and frozen in liquid nitrogen, and then stored at −80 °C. Phytohormone extraction and analysis were performed as previously reported with minor modifications (Alba *et al*., 2015). Briefly, frozen root samples were ground, and then 0.1 g powder was transformed into a 2 mL tube, which was filled with extracting solution H6‐ABA(8 ng mL^−1^). The homogenate was shaken and stored at 4 °C overnight to extract before centrifugation. The supernatant was collected, and the pellet was re‐extracted. The supernatants were combined and evaporated to dryness. The residue was resuspended in 100 μL of 40% (v/v) methanol and then centrifuged, filtered. The supernatants were analysed by LC–MS analysis on the Agilent 1290 infinity HPLC system.

## Funding information

We thank the National Natural Science Foundation of China (32201731), Outstanding Youth Foundation of He’nan Scientific Committee (222300420097), Innovation Program of Chinese Academy of Agricultural Sciences (CAAS‐CSIAF‐202402), Key Research and Development Task of Xinjiang Uygur Autonomous Region (2024B02006‐2), Supported by China Agriculture Research System of MOF and MARA (CARS‐15‐02) and the Fundamental Research Funds of State Key Laboratory of Cotton for financial support.

## Conflict of interest

The authors declare no competing interests.

## Author contributions

L.S. L., X.Y. G., H. Z. and Z.Z. X. supervised the experiments; X.Y. G., L.S. L., Z.Z. X. and J.H. M. designed the research; L.S. L., H.D. Z., F. J., Y. Y. and Y. W. performed the experiments; J.N. L., Y.L. C., Y. Y., J.J. Z. and H.Y. D. contributed materials and analysed data; L.S. L., X.Y. G., H. Z. and Z.Z. X. wrote the manuscript.

## Supporting information


**Figure S1** Suppression of Vd424Y‐triggered cell death by Vd06254. (A) Vd06254 suppresses Vd424Y‐triggered cell death in *Nicotinana benthamiana*. Four‐week‐old plants were used to express *Vd06254* within the regions indicated by dashed lines, at 24 h post‐infiltration with *Vd424Y*. (B) Western blot analysis of protein levels using anti‐HA antibodies. Proteins gels were stained with Coomassie Brilliant R‐250 (CBB) to confirm equal loading.
**Figure S2** Suppression of Vd424Y‐regulated immune‐associated genes by Vd06254 in *Nicotiana benthamiana* at 3 days post‐infiltration (dpi). Relative expression of immune‐associated marker genes in *N. benthamiana* infiltrated with *Agrobacterium tumefaciens* carrying Vd424Y, Vd06254 and Vd06254 + Vd424Y. At 3 dpi, total RNA was extracted and transcript levels were detected by quantitative reverse transcriptase PCR (RT‐qPCR). *NbActin* was used as the internal reference gene. The data shown represents the mean across three independent experiments. Bars indicate standard error (SE, n = 3). Significance levels *P* < 0.05, 0.01, and 0.001 are represented by *, ** and ***, respectively.
**Figure S3** Identification of transgenic cotton plants stably expressing *Vd06254 or Vd06254* ^*ΔNLS*^. Reverse transcriptase PCR (RT‐PCR) analysis of *Vd06254* or *Vd06254* ^*ΔNLS*^ mRNA levels in the transgenic lines and wild‐type (WT) plants.
**Figure S4** Knockout of *Vd06254* using targeted gene replacement and gene complementation. (a) Organization of *Vd06254* locus before and after homologous recombination in wild‐type Vd991. (b) PCR analysis of wild‐type Vd991 and mutants. The genomic DNA of each strain was used to verify the targeted gene and hygromycin resistance gene (*HPH*) gene.
**Figure S5** Quantitative reverse transcriptase PCR (RT‐qPCR) analysis of Vd06254‐regulated immune‐associated genes in cotton roots infected by Vd991 or *ΔVd06254* at 12 h post‐infiltration (hpi). Relative expression levels of immune‐associated marker genes in cotton were normalized to the control gene *GhActin*. The data shown as means ± standard error (SE) (*n* = 3). Significance levels *P* < 0.05, 0.01 and 0.001 are represented by *, ** and ***, respectively.
**Figure S6** Subcellular localization of GhMYC3‐YFP, Vd06254‐YFP and Vd06254 ^ΔNLS^‐YFP in *N. benthamiana* leaves via *Agrobacterium*‐mediated transient expression. Fluorescence was detected in epidermal cells of infiltrated leaf tissues at 48 h post‐infiltration based on confocal microscopy. Scale bars: 50 μm.
**Figure S7** Sanger sequencing in wild‐type cotton Baimian 1 and *GhMYC3*‐edited plants confirming the knockout of *GhMYC3*.
**Figure S8** Transcriptional activation assay in the *N. benthamiana* leaves via *Agrobacterium*‐mediated transient expression showed that the transcription of *GhCCD8* was suppressed by GhMYC3. Values are mean ± standard deviation. * and ** indicates a significant difference at a *P*‐value of <0.05 and 0.01, respectively.
**Figure S9** Concentrations of strigolactones (SL) in the roots of ZZM2 plants treated with and without *Verticillium dahliae*.
**Figure S10** Exogenous application of rac‐GR24 enhances cotton resistance to *Verticillium dahliae*.
**Figure S11** The expression pattern of *GhCCD8* in leaves of ZM24 after the application of 10 μM of jasmonic acid (JA) and the expression level of *GhMYC3* in wild‐type (WT), *OE‐GhCCD8* and *KO‐GhCCD8* treated without or with *Verticillium dahliae*.
**Figure S12** *GhCCD8* promotes SL and JA synthesis and positively regulates SL‐ and JA‐signalling pathway.
**Figure S13** *GhMYC3* promotes JA synthesis and regulates JA‐signalling pathway, while represses SL synthesis and down‐regulates SL‐signalling pathway.
**Figure S14** Sequence alignment between GbMYC2 and GhMYC3.

**Table S1** Primers used in this study.

## Data Availability

All data included in this study are available.
