# Supplementary material for: Verticillium dahliae effector Vd06254 disrupts cotton defence response by interfering with GhMYC3‐ GhCCD8 ‐mediated hormonal crosstalk between jasmonic acid and strigolactones
Source: Plant Biotechnol J. 2025 Apr 22;23(7):2755–68. doi: 10.1111/pbi.70098 (PMC12205861; doi:10.1111/pbi.70098)
Supplement: Supplementary file 1 — Figure S1 Suppression of Vd424Y‐triggered cell death by Vd06254. (A) Vd06254 suppresses Vd424Y‐triggered cell death in Nicotinana benthamiana. Four‐week‐old plants were used to express Vd06254 within the regions indicated by dashed lines, at 24 h post‐infiltration with Vd424Y. (B) Western blot analysis of protein levels using anti‐HA antibodies. Proteins gels were stained with Coomassie Brilliant R‐250 (CBB) to confirm equal loading. Figure S2 Suppression of Vd424Y‐regulated immune‐associated genes by Vd06254 in Nicotiana benthamiana at 3 days post‐infiltration (dpi). Relative expression of immune‐associated marker genes in N. benthamiana infiltrated with Agrobacterium tumefaciens carrying Vd424Y, Vd06254 and Vd06254 + Vd424Y. At 3 dpi, total RNA was extracted and transcript levels were detected by quantitative reverse transcriptase PCR (RT‐qPCR). NbActin was used as the internal reference gene. The data shown represents the mean across three independent experiments. Bars indicate standard error (SE, n = 3). Significance levels P < 0.05, 0.01, and 0.001 are represented by *, ** and ***, respectively. Figure S3 Identification of transgenic cotton plants stably expressing Vd06254 or Vd06254 ΔNLS . Reverse transcriptase PCR (RT‐PCR) analysis of Vd06254 or Vd06254 ΔNLS mRNA levels in the transgenic lines and wild‐type (WT) plants. Figure S4 Knockout of Vd06254 using targeted gene replacement and gene complementation. (a) Organization of Vd06254 locus before and after homologous recombination in wild‐type Vd991. (b) PCR analysis of wild‐type Vd991 and mutants. The genomic DNA of each strain was used to verify the targeted gene and hygromycin resistance gene (HPH) gene. Figure S5 Quantitative reverse transcriptase PCR (RT‐qPCR) analysis of Vd06254‐regulated immune‐associated genes in cotton roots infected by Vd991 or ΔVd06254 at 12 h post‐infiltration (hpi). Relative expression levels of immune‐associated marker genes in cotton were normalized to the control gene GhActin. [file PBI-23-2755-s002.docx]

**Supporting Information**


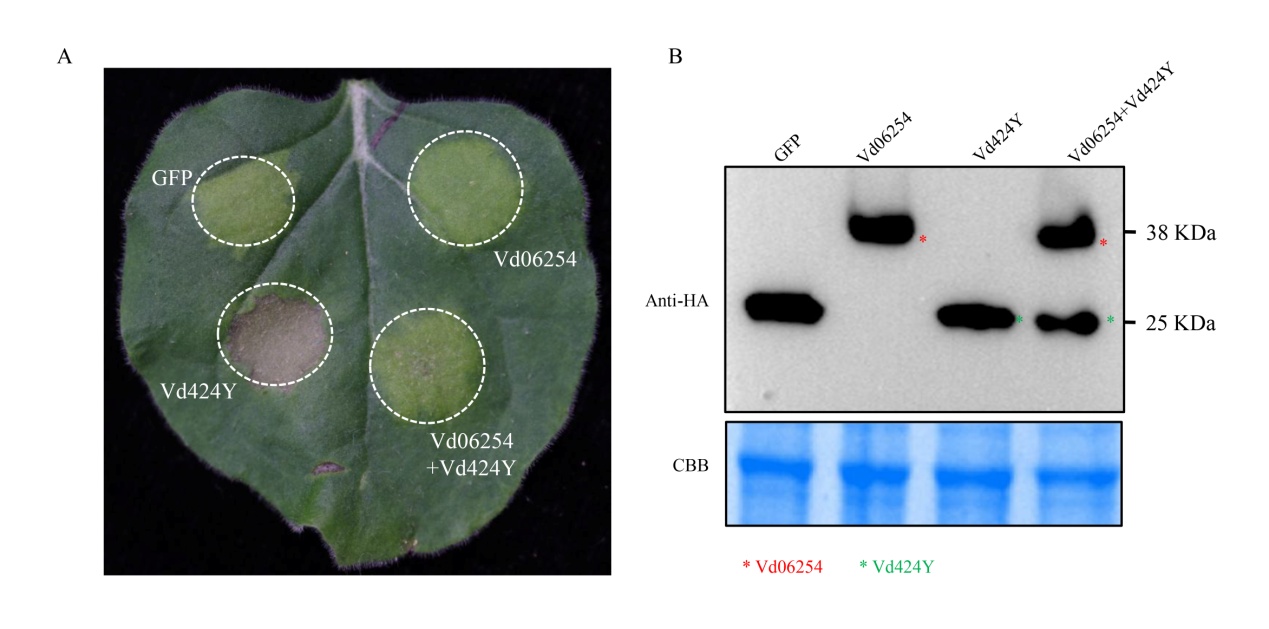


**Figure S1.** **Suppression of Vd424Y-triggered cell death by Vd06254.** (A) Vd06254 suppresses Vd424Y-triggered cell death in *Nicotiana benthamiana*. Four-week-old plants were used to express *Vd06254* within the regions indicated by dashed lines, at 24 hours post-infiltration (hpi) with *Vd424Y*. (B) Western blot analysis of protein levels using anti-HA antibodies. Proteins gels were stained with Coomassie Brilliant R-250 (CBB) to confirm equal loading.


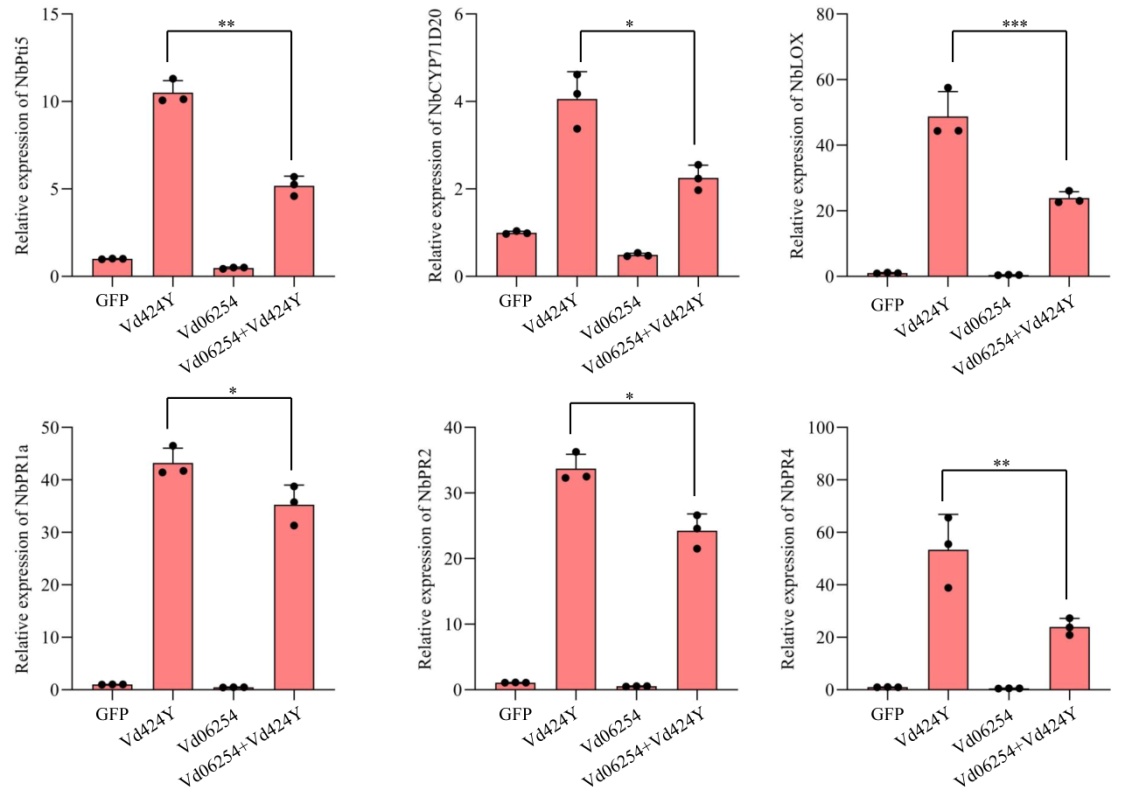


**Figure S2.** **Suppression of Vd424Y-regulated immune-associated genes by Vd06254 in *Nicotiana benthamiana* at 3 days post-infiltration.** Relative expression of immune-associated marker genes in *Nicotiana benthamiana* infiltrated with Agrobacterium tumefaciens carrying Vd424Y, Vd06254, and Vd06254+Vd424Y. At 3 days post-infiltration (dpi), total RNA was extracted and transcript levels were assessed by quantitative reverse transcriptase PCR (RT-qPCR). *NbActin* served as the internal reference gene. The data shown represents the mean across three independent experiments. Bars indicate standard error (SE, n = 3). Significance levels *P* <0.05, 0.01, and 0.001 are represented by *, **, and ***, respectively.


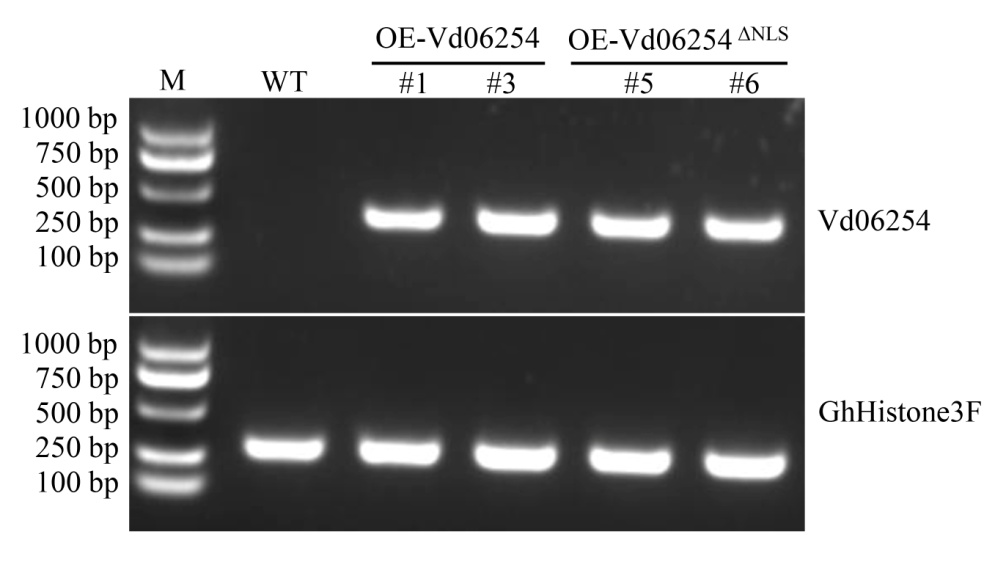


**Figure S3.** **Identification of transgenic cotton plants stably expressing *Vd06254* or *Vd06254 ^ΔNLS^*.** Reverse transcriptase PCR (RT-PCR) analysis of *Vd06254* or *Vd06254 ^ΔNLS^* mRNA levels in transgenic lines compared to wild-type (WT) plants.


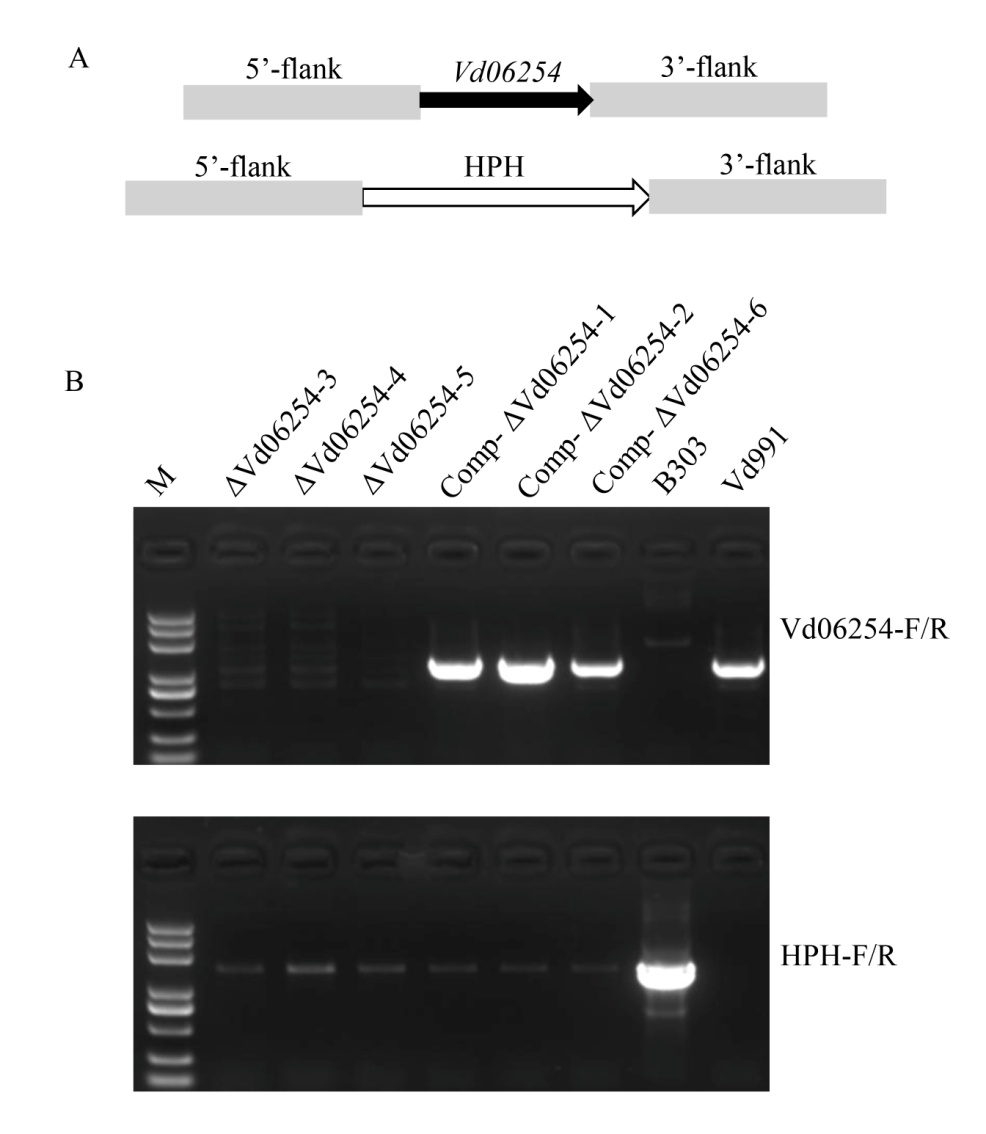


**Figure S4.** **Knockout of *Vd06254* by targeted gene replacement and gene complementation.** (**A**) Organization of *Vd06254* locus before and after homologous recombination in wild-type (WT) Vd991. (**B**) PCR analysis of WT Vd991 and mutants. The genomic DNA of each strain was used to verify the targeted gene and hygromycin resistance gene (*HPH*) gene. B303, a positive control containing HPH gene.


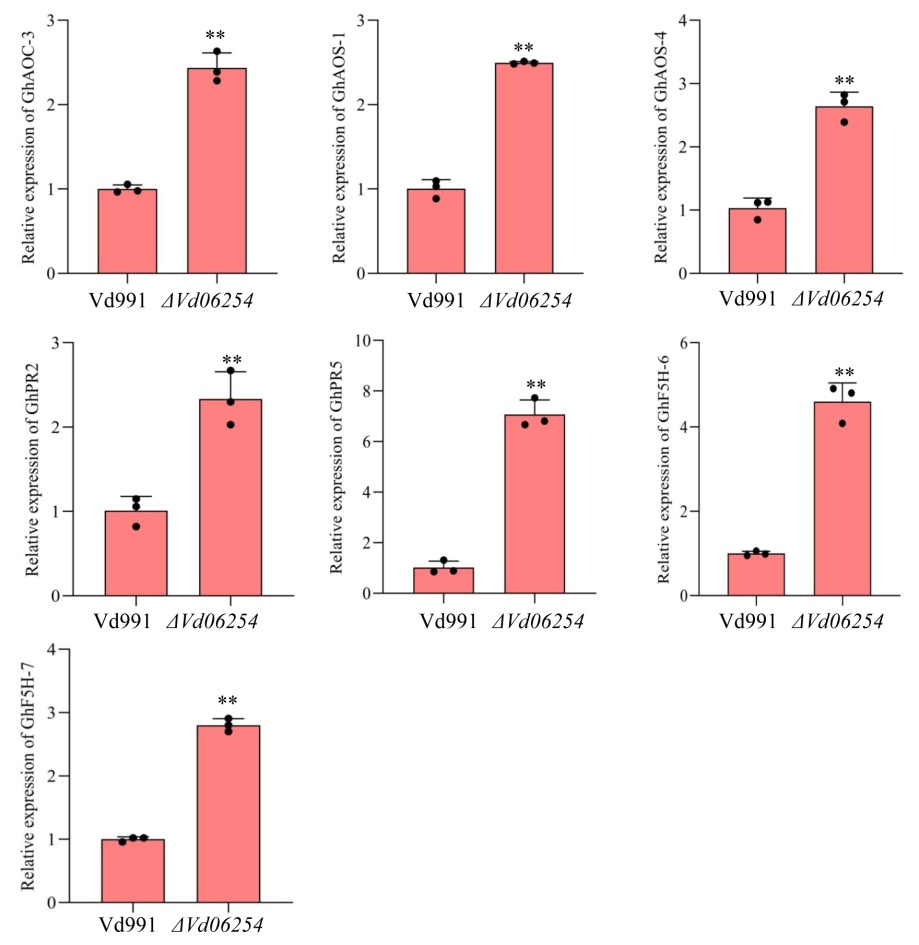


**Figure S5.** Quantitative reverse transcriptase PCR (RT-qPCR) analysis of Vd06254-regulated immune-associated genes in cotton roots infected by Vd991 or *ΔVd06254* at 12 hours post-inoculation (hpi). Relative expression levels of immune-associated marker genes in cotton were normalized to the control gene *GhActin*. The data were shown as means ± SE (n = 3). Significance levels *P* <0.05, 0.01, and 0.001 are represented by *, **, and ***, respectively.


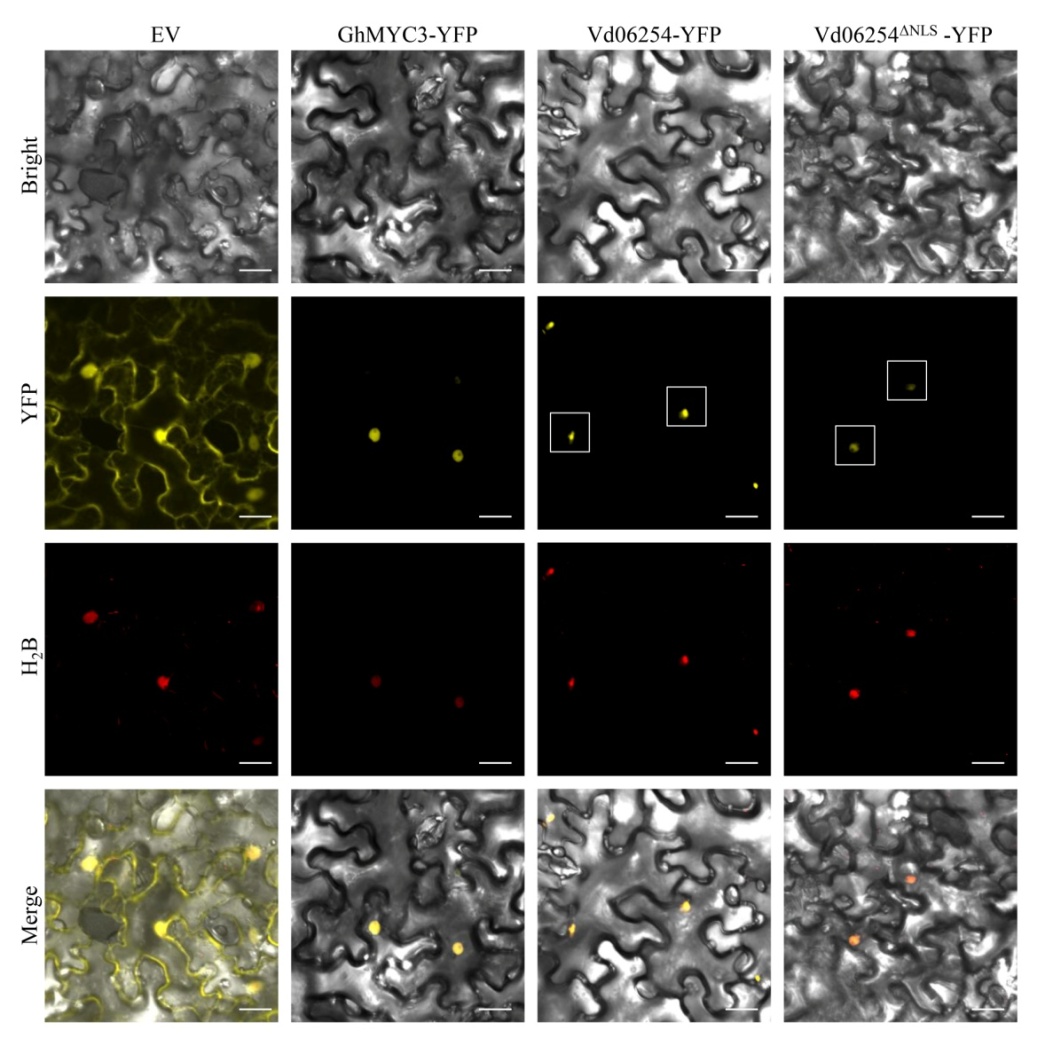


**Figure S6.** Subcellular localization of GhMYC3-YFP, Vd06254-YFP, and Vd06254 ^ΔNLS^-YFP in *N. benthamiana* leaves via *Agrobacterium*-mediated transient expression. Fluorescence was detected in epidermal cells of infiltrated leaf tissues at 48 h post-infiltration based on confocal microscopy. Scale bars: 50 µm.


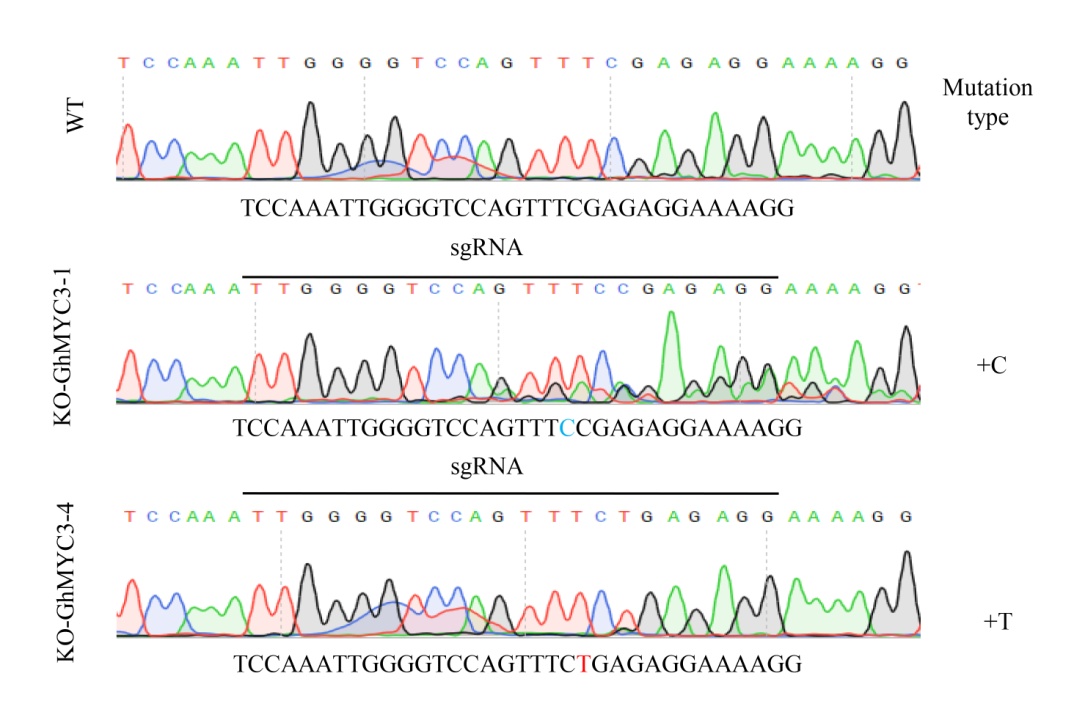


**Figure S7.** Genetic characterization of wild-type (WT) Baimian 1 cotton and GhMYC3-edited plants using Sanger sequencing.


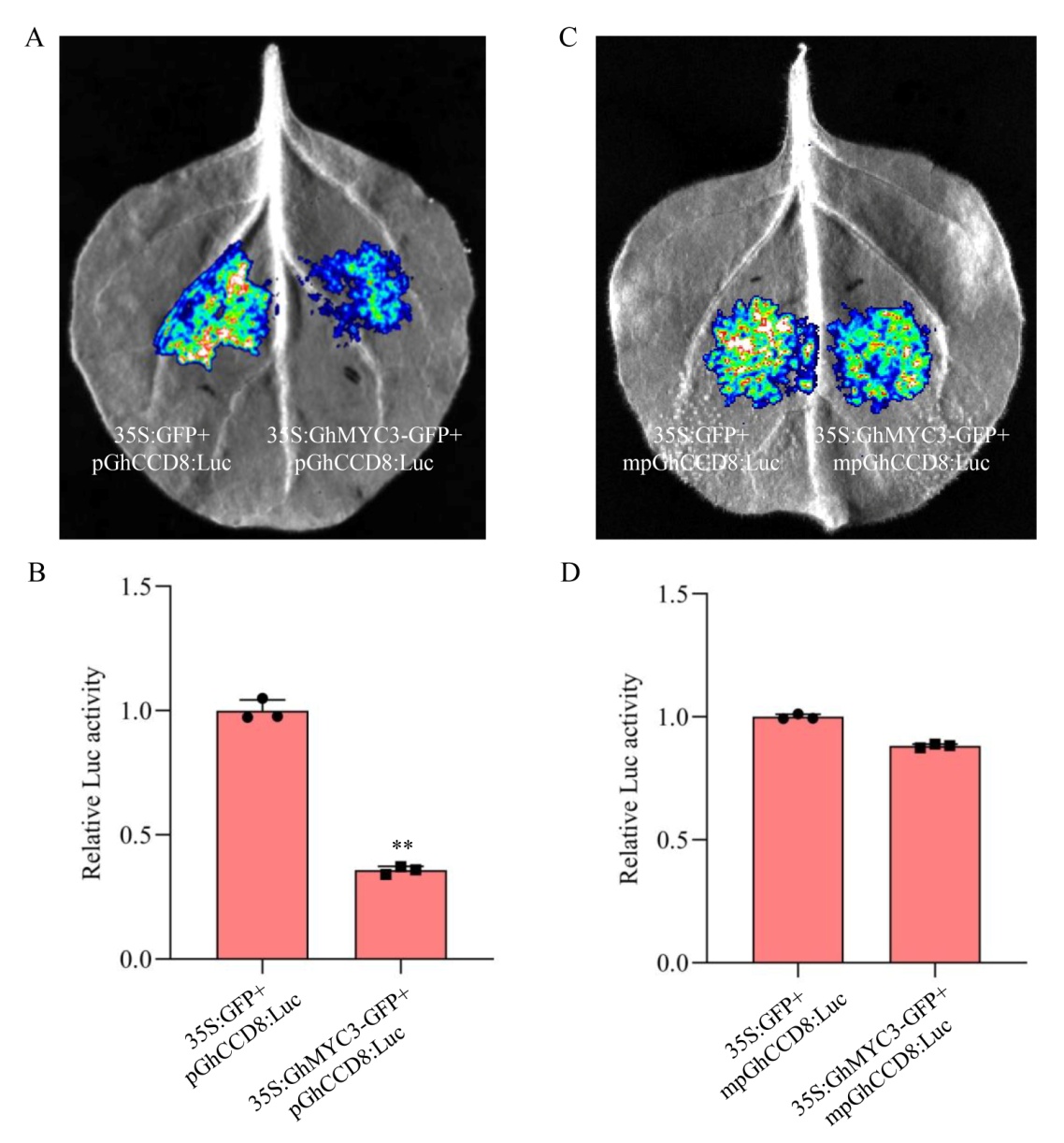


**Figure S8.** Transcriptional activation assay in the *N. benthamiana* leaves via *Agrobacterium*-mediated transient expression showed that the transcription of *GhCCD8* was suppressed by GhMYC3. Values are mean ± standard deviation. * and ** indicates a significant difference at a *P*-value of < 0.05 and 0.01, respectively.


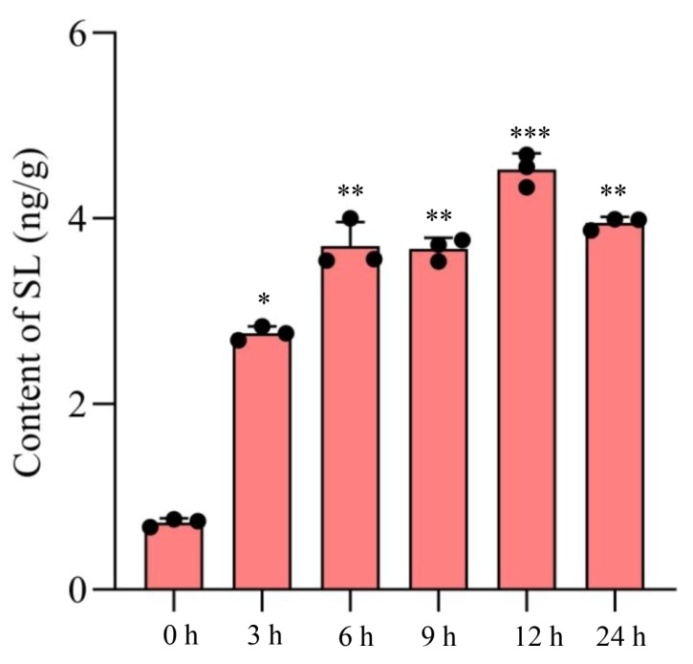


**Figure S9.** Concentrations of SL in roots of ZZM2 plants treated with and without *Verticillium dahliae*. The data shown as means ±standard error (SE, n = 3). Significance levels at *P* <0.05, 0.01, and 0.001 are represented by *, **, and ***, respectively.


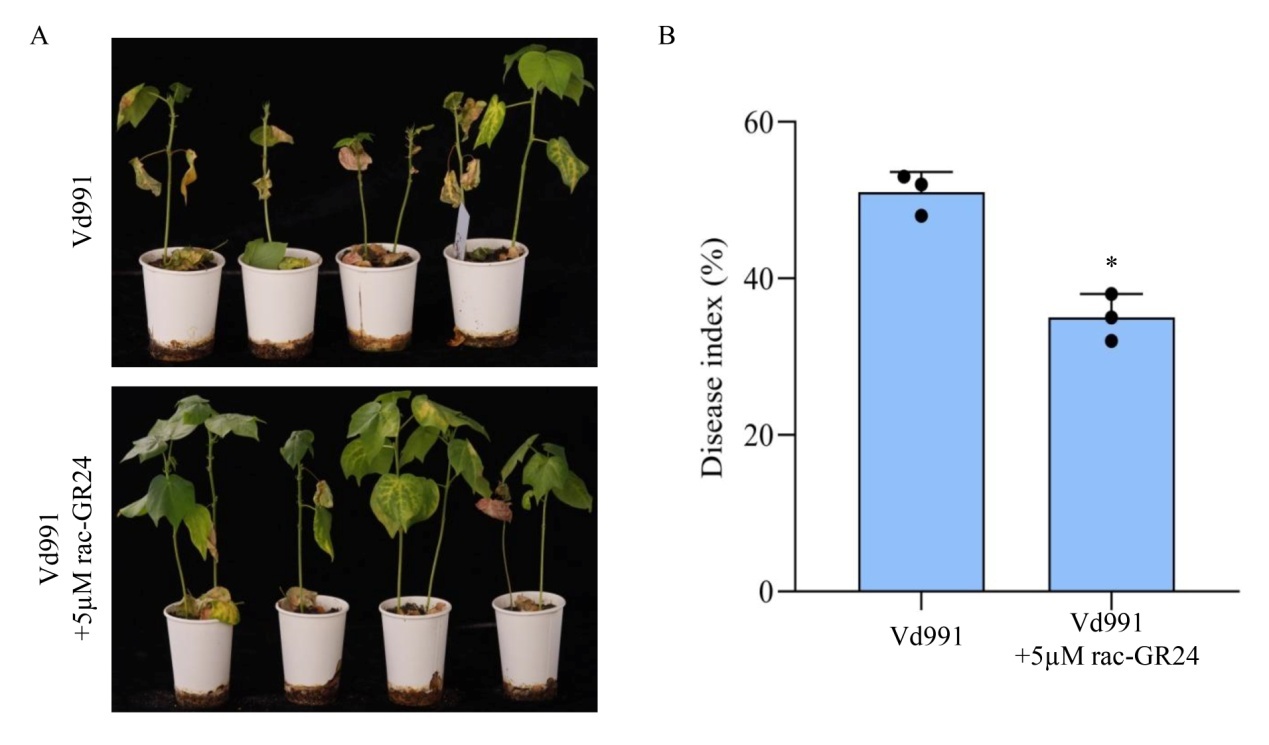


**Figure S10.** Exogenous application of rac-GR24 enhances cotton resistance to *Verticillium dahliae*. **(A)** Disease symptoms of ZM24 (susceptible cotton cultivar) plants sprayed with rac-GR24 (5 µM) or Mock control (water containing an equal amount of ethanol) plants for 24 hours post-inoculation (hpi) with *V. dahliae* spores. The application of rac-GR24 was conducted twice per week during the infection stage to maintain the effect of rac-GR24. **(B)** Disease index (DI) of ZM24 cotton plants at 30 days post-inoculation (dpi) and rac-GR24 treatment. Data are presented as means ±standard deviation (SD) of three biological replicates (n = 30). *, *P* < 0.05.


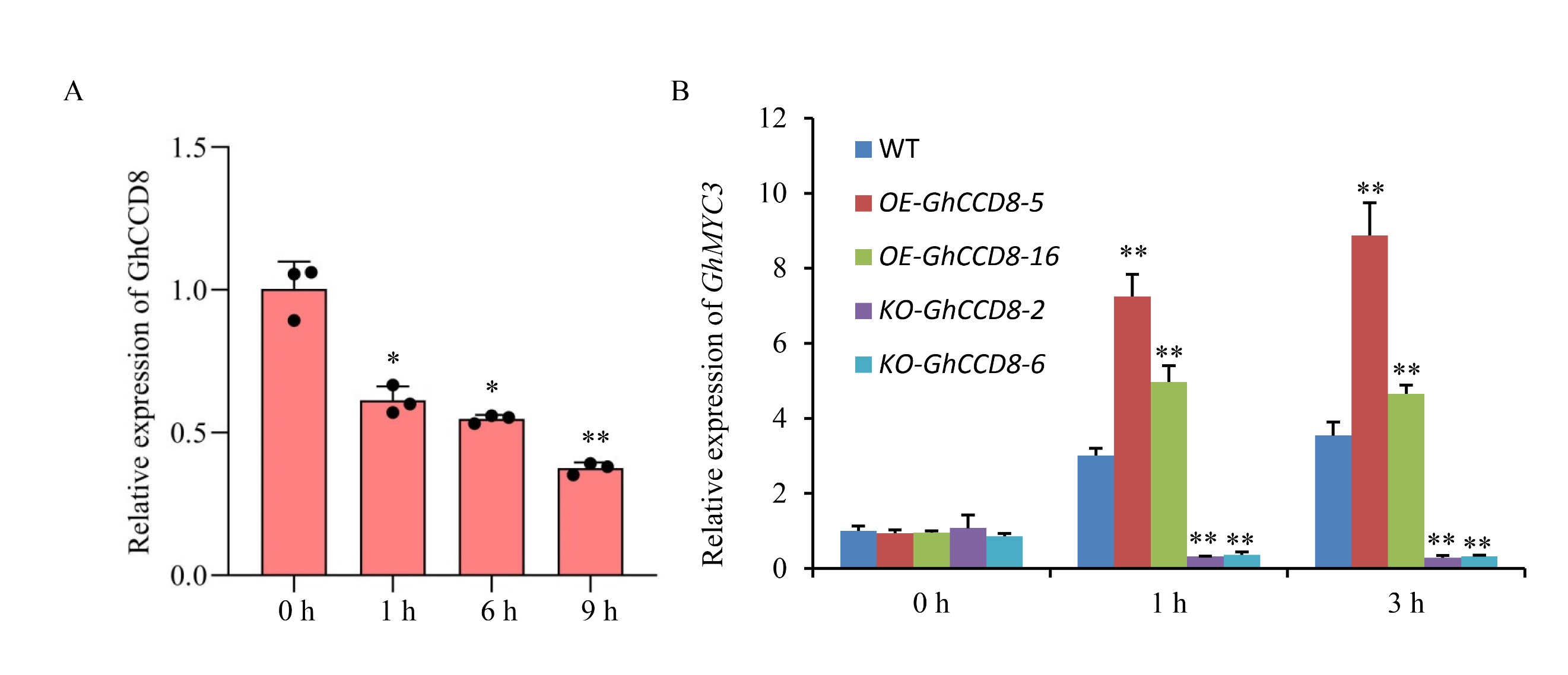


**Figure S11.** The expression pattern of *GhCCD8* in leaves of ZM24 after the application of 10 µM of jasmonic acid (JA) and the expression level of *GhMYC3* in wild-type (WT), *OE-GhCCD8*, and *KO-GhCCD8* treated with or without *Verticillium dahliae*. (**A**) Relative transcript levels of *GhCCD8* in ZM24 plants after treatment with 10 µM of JA for 0, 1, 6, and 9 hours (h). The expression value at 0 h was normalized as 1. (**B**) Expression analysis of *GhMYC3* in WT, *OE-GhCCD8*, and *KO-GhCCD8* cotton plants inoculated with and without *V. dahliae*, respectively. The data shown as means ± standard error (SE, n = 3). Significance levels at *P* <0.05, and 0.01 are represented by *, and *, respectively.


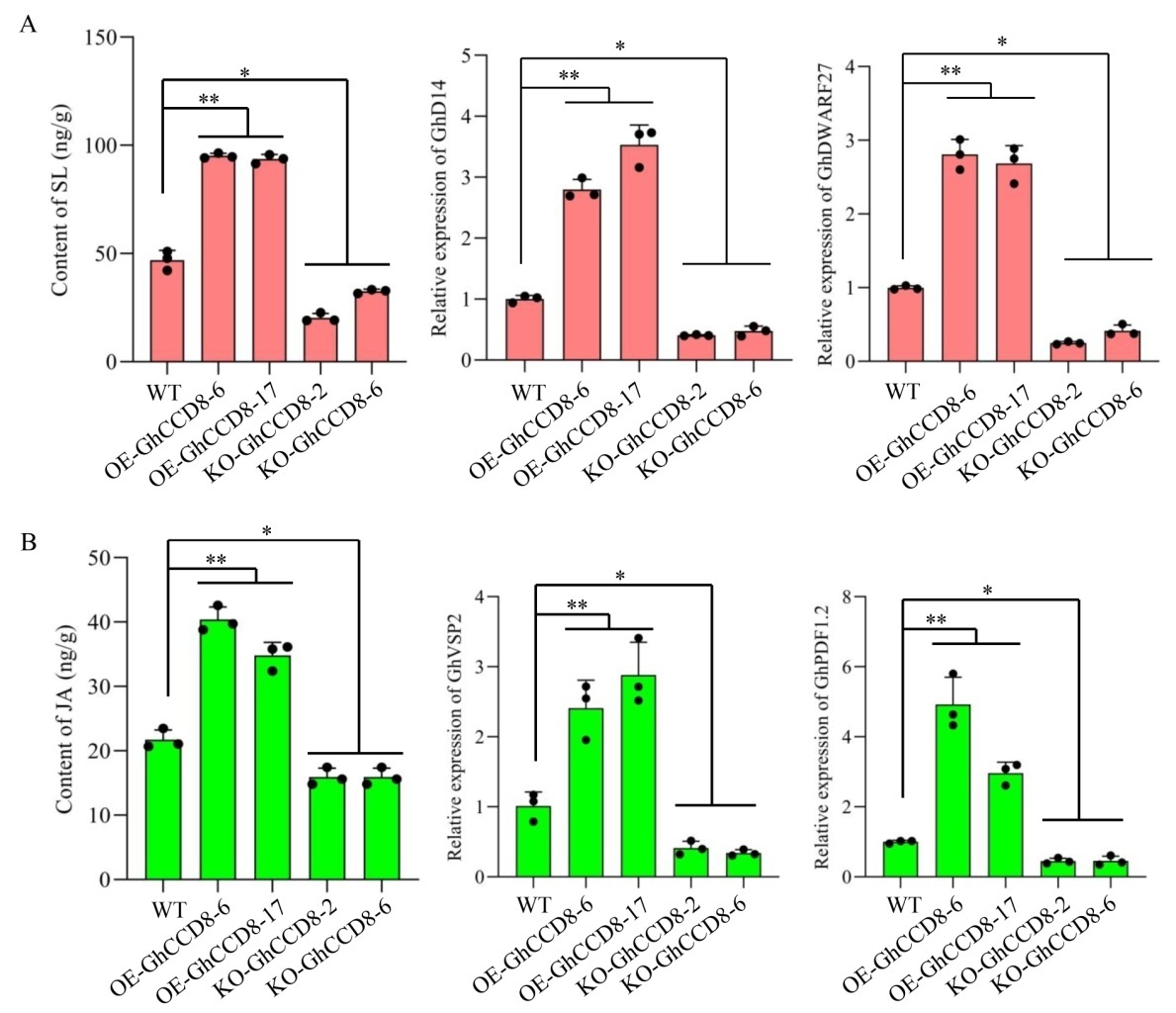


**Figure S12.** ***GhCCD8* promotes SL and JA synthesis, and positively regulates SL- and JA-signaling pathway. (A)** Concentration of SL in roots and SL signaling pathway marker genes expression in leaves of WT, OE-GhCCD8, and KO-GhCCD8 plants. **(B)** Concentration of JA in roots and JA signaling pathway marker genes expression in leaves of WT, OE-GhCCD8, and KO-GhCCD8 plants. The data shown as means ± standard error (SE, n = 3). Significance levels at *P* <0.05, and 0.01 are represented by *, and *, respectively.


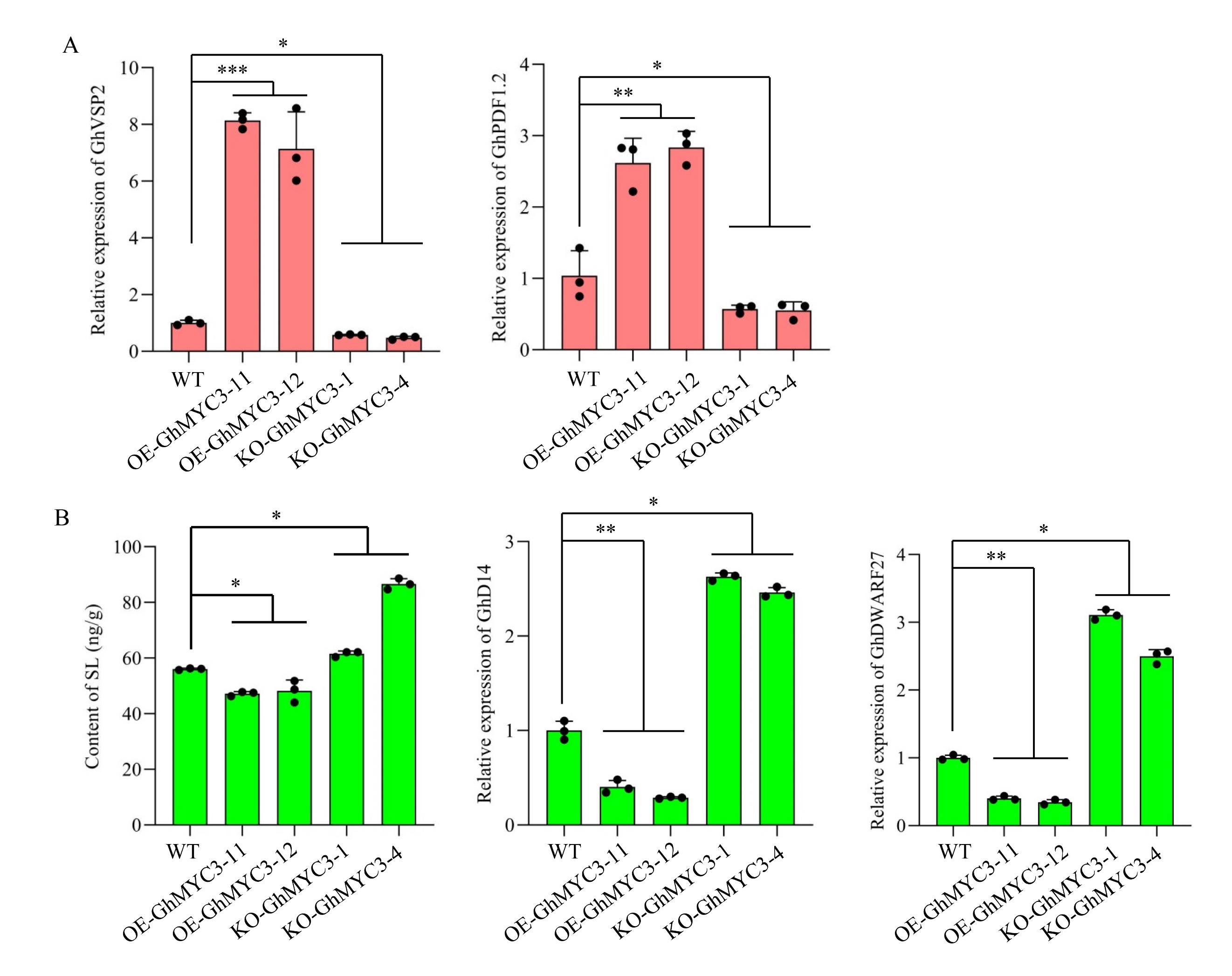


**Figure S13.** ***GhMYC3* promotes JA synthesis and regulates JA-signaling pathway, while represses SL synthesis and down-regulates SL-signaling pathway. (A)** JA signaling pathway marker genes expression in leaves of WT, OE-GhMYC3, and KO-GhMYC3 plants. **(B)** Concentration of SL in roots and SL signaling pathway marker genes expression in leaves of WT, OE-GhMYC3, and KO-GhMYC3 plants. The data shown as means ± standard error (SE, n = 3). Significance levels at *P* <0.05, and 0.01 are represented by *, and *, respectively.


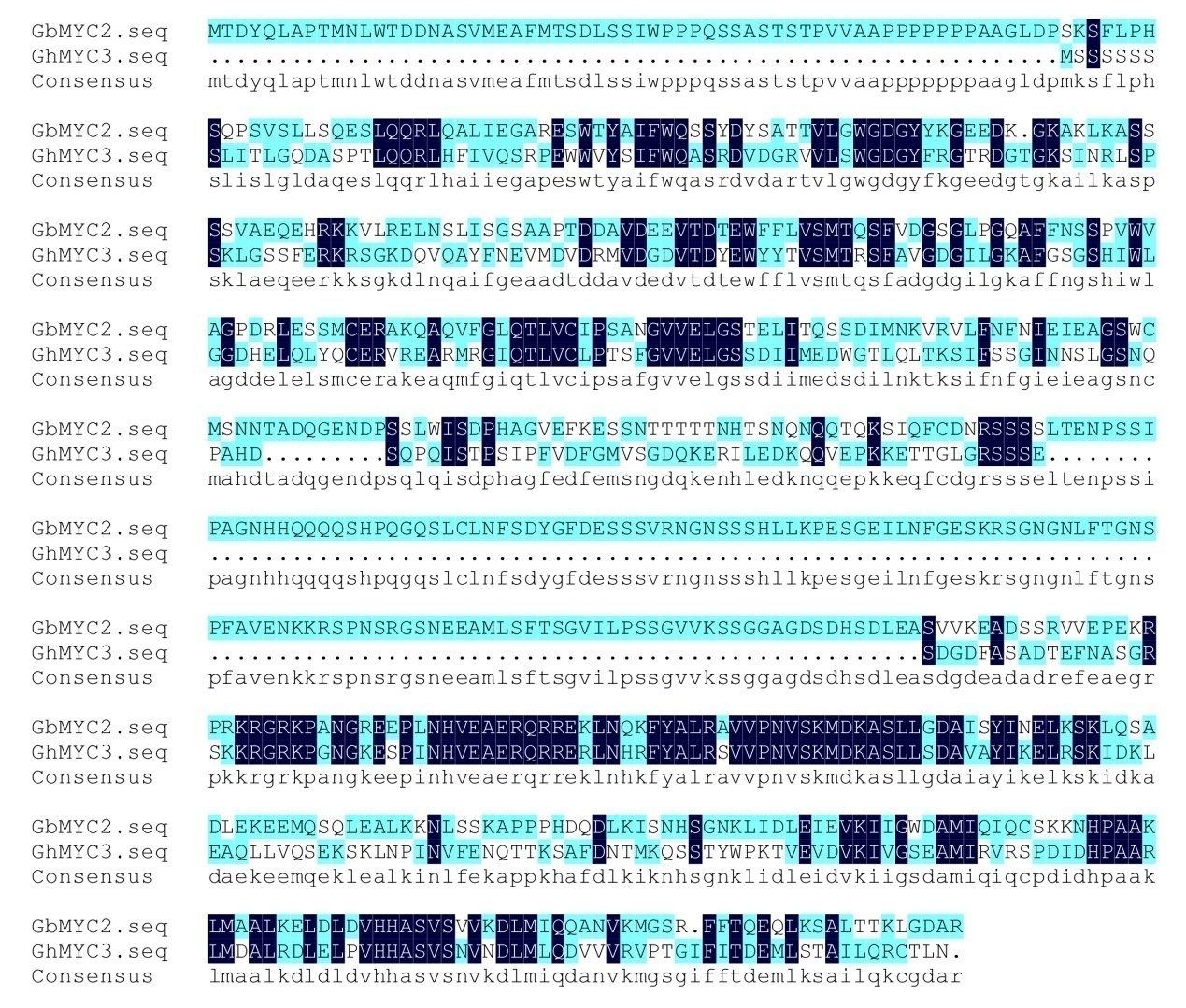


**Figure S14.** Sequence alignment between GbMYC2 and GhMYC3.
